# Development of a Nanobody-based lateral flow assay to detect active *Trypanosoma congolense* infections

**DOI:** 10.1038/s41598-018-26732-7

**Published:** 2018-06-13

**Authors:** Joar E. Pinto Torres, Julie Goossens, Jianzu Ding, Zeng Li, Shaohong Lu, Didier Vertommen, Peter Naniima, Rui Chen, Serge Muyldermans, Yann G.-J. Sterckx, Stefan Magez

**Affiliations:** 1 Research Unit for Cellular and Molecular Immunology (CMIM), VUB, Pleinlaan 2, B-1050 Brussels, Belgium; 2 Structural Biology Research Center (SBRC), VIB, Pleinlaan 2, B-1050 Brussels, Belgium; 3 Institute of Parasitic Diseases, Zhejiang Academy of Medical Sciences, Hangzhou, 310013 Zhejiang China; 4 Department of Metabolism and Hormones, de Duve Institute, Université Catholique de Louvain, Avenue Hippocrate 75, B-1200 Brussels, Belgium; 5 Ghent University Global Campus, Songdomunhwa-Ro 119, Yeonsu-Gu, 406-840 Incheon, South Korea; 6 Institute of Virology, Structural Virology Group, Medizinische Hochschule Hannover, Carl-Neuberg-Strasse 1, 30625 Hannover, Germany

## Abstract

Animal African trypanosomosis (AAT), a disease affecting livestock, is caused by parasites of the *Trypanosoma* genus (mainly *T*. *vivax* and *T*. *congolense*). AAT is widespread in Sub-Saharan Africa, where it continues to impose a heavy socio-economic burden as it renders development of sustainable livestock rearing very strenuous. Active case-finding and the identification of infected animals prior to initiation of drug treatment requires the availability of sensitive and specific diagnostic tests. In this paper, we describe the development of two heterologous sandwich assay formats (ELISA and LFA) for *T*. *congolense* detection through the use of Nanobodies (Nbs). The immunisation of an alpaca with a secretome mix from two *T*. *congolense* strains resulted in the identification of a Nb pair (Nb44/Nb42) that specifically targets the glycolytic enzyme pyruvate kinase. We demonstrate that the Nb44/Nb42 ELISA and LFA can be employed to detect parasitaemia in plasma samples from experimentally infected mice and cattle and, additionally, that they can serve as ‘test-of-cure’ tools. Altogether, the findings in this paper present the development and evaluation of the first Nb-based antigen detection LFA to identify active *T*. *congolense* infections.

## Introduction

Animal African trypanosomosis (AAT) is a neglected tropical disease caused by parasites of the *Trypanosoma* genus and mainly affects livestock in Sub-Saharan Africa. AAT is hallmarked by a mild to severe pathology in large and small ruminants, which results in drastic reductions of draft power, meat and milk production by the infected animals. It is believed that approximately 50 million livestock animals across Sub-Saharan Africa are currently at risk of infection. Thus, AAT has both direct and indirect consequences on the socio-economic development of the endemic areas with yearly losses estimated in the order of magnitude of billion USD^1,2^. The most important causative *Trypanosoma* species for AAT are *T*. *congolense*, *T*. *vivax* and, to a lesser extent, *T*. *brucei brucei*^3,4^. In many rural regions, the prevalence of *T*. *congolense* and *T*. *vivax* infections is prominent (20–40%) and mixed infections have been reported to be quite common^5,6^. Prevention of AAT requires the concerted action of vector control programs^7,8^ and drug treatment schemes^9^, especially since vaccine development experiences major hurdles^10,11^. The success of prevention and control initiatives heavily depends on the availability of accurate, sensitive and specific diagnostic methods. Unfortunately, most tests currently employed in endemic areas do not meet the required standards. In many cases, cattle are treated based on symptoms, which have the problem of not being pathognomonic. While the situation has been improved by the advent of microscopic methods such as the hematocrit technique^12,13^, the sensitivity and specificity of these assays remain relatively low. The lack of routine field diagnosis has resulted in the indiscriminate use (and misuse) of trypanocidal drugs, thereby accelerating the rise of parasite resistance^14–16^. The situation is further complicated by the circulation of counterfeit products^17^ and drug administration by farmers themselves because of limited veterinary assistance^18^.

Within a healthcare context, the development of so-called ‘rapid diagnostic tests (RDTs)’ is of primary interest. RDTs are diagnostic assays from which the results are available within a short time such that control and treatment strategies may be initiated as soon as possible. The ideal RDT to be used under field conditions has to meet the requirements of the ‘ASSURED’ principle: *a*ffordable by those at risk of infection, *s*ensitive, *s*pecific, *u*ser-friendly, *r*apid and robust, *e*quipment-free, and *d*elivered to those in need^19^. Diagnosis of trypanosomes is either based on the detection of parasite molecules (DNA and protein) or host antibodies against parasite antigens. Recently, the sensitivity and specificity of AAT diagnosis have greatly been improved by the development of DNA-based assays such as PCR and LAMP, which aim to amplify parasite DNA^20–24^. While such assays are highly reliable and have the potential of detecting an infection before the manifestation of pathology, their deployment in the field is generally difficult. Indeed, PCR requires a laboratory environment and trained personal and LAMP could be prone to contamination during sample preparation in the field^25,26^. Furthermore, it is has been documented that livestock rearing communities heavily burdened by AAT rarely call upon trained personnel to perform diagnosis and drug treatment^27^. Hence, DNA-based diagnostic tests do not yet meet all ASSURED criteria. Serological immunoassays (such as enzyme-linked immunosorbent assays; ELISA), which either detect host antibodies against parasite antigens (antibody-based assays) or parasite antigens directly (antigen-based assays), may represent interesting starting points for RDT development. These rely on the formation of specific antibody-antigen complexes and can be translated into lateral flow assays (LFAs).

Both antibody- and antigen-based formats have their advantages and drawbacks. Antibody-based assays, of which the sensitivity relies on the magnitude of the host’s humoral response, have two potential flaws: (i) a low specificity due to antibody cross-reactivity^28^, and (ii) the occurrence of long lasting antibodies that remain in circulation weeks to months after clearance of infection, thereby making the identification of active infections unreliable^6,29,30^. Several antibody-based immunoassays have been developed for *T*. *congolense* and *T*. *vivax*^31–35^. While some of them have been successfully deployed in large-scale surveys^31,32,35^, one has recently been commercialised^35,36^. *T*. *congolense* specific antigen-based ELISAs have also been described^37,38^. Although antigen-based immunoassays do not experience the aforementioned issues, they are not flawless. Since the sensitivity of antigen-based assays is mainly determined by the amount of circulating parasite antigen, the latter should be high enough to be detected^39^. Additionally, the target antigen should possess epitopes specific to a given parasite genus or even species. The capturing and detecting antibodies constituting the assay may either recognise the same (homologous) or distinct (heterologous) epitopes, thereby imposing additional requirements on the nature of the antigen depending on the used set-up: while the antigen does not have to be a multimer or monomer with repeating epitopes for detection in heterologous systems, this is a necessity in the case of homologous antigen-based assays^40–42^. Also, the assay’s antibodies must be able to outcompete host antibodies or bind different epitopes^39^ to avoid false negative scores and must not interact directly with host anti-IgG antibodies to avoid false positive signals (this occurs generally through interactions with the constant Fc domains^43^). These pitfalls could be avoided by the use of Nanobodies (Nbs) as primary reagents in the immunoassays. Nbs are small antigen-binding entities (15 kDa) corresponding to the variable domains of camelid heavy-chain only antibodies^44,45^. First, Nbs have the potential to recognise epitopes distinct from those targeted by conventional antibodies^46,47^. Second, these single-domain antibody fragments do not possess an Fc domain, thereby reducing the likelihood of non-specific interactions with host antibodies. Finally, Nbs can easily be tailored into multivalent and/or tagged constructs^48^ and can be coated onto gold nanoparticles (AuNPs)^41,49^, which adds to their versatility as detection tools for LFA use.

The application of a Nb-based sandwich ELISA for the specific detection of *T*. *congolense* infections has recently been described^42^. Despite the assay’s decent performance on fresh field samples (sensitivity and specificity of 87% [95% CI, 73–95%] and 94% [95% CI, 71–100%], respectively), its translation into an LFA will be difficult due to its homologous set-up. In this paper, we describe the development of a Nb-based *T*. *congolense* specific heterologous sandwich ELISA and its translation into an LFA. Two Nbs, Nb42 and Nb44, were identified by immunising an alpaca with *T*. *congolense* secretome, which constitutes the proteome fraction that is actively secreted by the parasite in the host’s blood stream. We demonstrate that the Nb44/Nb42 pair specifically recognises *T*. *congolense* pyruvate kinase (*Tco*PYK), thereby validating yet another glycolytic enzyme as a candidate biomarker. Using these Nbs, we have developed antigen-based immunoassays in two formats: a heterologous sandwich ELISA and an LFA prototype. We discuss assay optimisation and show that both can be used as test-of-cure tools in experimental infections in mice. Finally, we present a preliminary evaluation of the performance of the LFA on plasma samples from experimentally infected cattle.

## Results

### An anti-*T*. *congolense* secretome Nb library yields a Nb pair (Nb44/Nb42) specific for *T*. *congolense*

**Figure 1:**
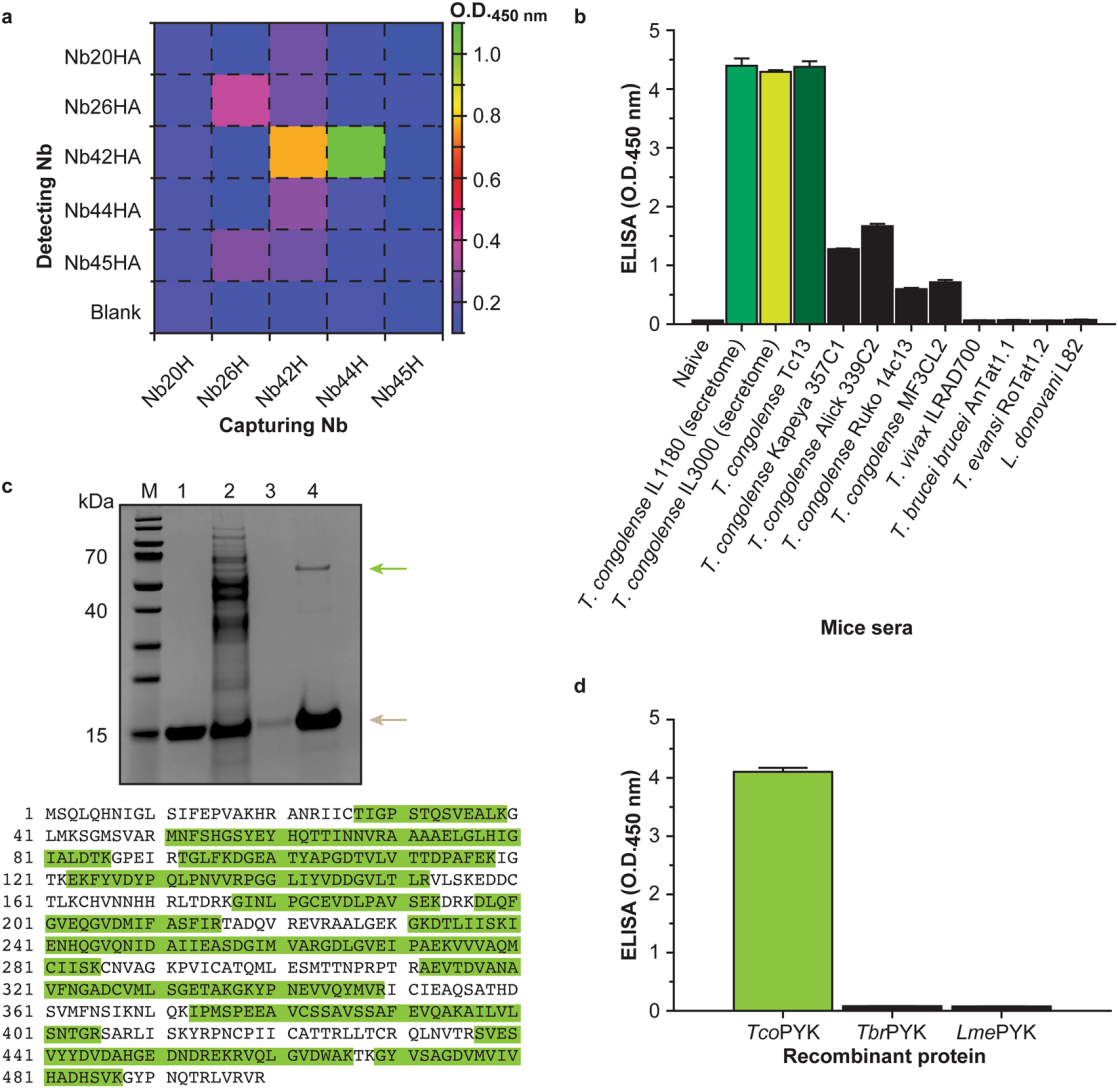
An anti-*T*. *congolense* secretome Nb library yields a Nb pair (Nb44/Nb42) that targets *T*. *congolense* pyruvate kinase. (**a**) Heat map representation of the results of the sandwich ELISA pairing assay performed with the five Nbs retrieved from the *T*. *congolense* secretome library (Nb20, Nb25, Nb42, Nb44 and Nb45). In this set-up, the capturing Nbs carry a C-terminal hexahistidine tag (denoted by the suffix ‘H’), while the detecting Nbs harbour a haemagglutinin tag followed by a hexahistidine tag at their C-terminus (denoted by the suffix ‘HA’). (**b**) Heterologous sandwich ELISA using the Nb44/Nb42 pair to determine its specificity. The sera from mice infected with various *Trypanosoma* and one *Leishmania* species were tested. *T*. *congolense* secretome samples and naive mouse sera were employed as positive and negative controls, respectively. (**c**) The top panel displays the Nb42H immuno-capturing experiment. *T*. *congolense* secretome was allowed to interact with nickel beads loaded with Nb42H. After a washing step, the captured protein was eluted and analysed on a 10% SDS-PAGE developed with Coomassie blue. Lane M, Prestained Protein Molecular Weight Marker (ThermoFischer Scientific); Lane 1, Nb42H; Lane 2, secretome flow through; Lane 3, wash; Lane 4, eluted protein. Native *Tco*PYK and Nb42H migrate at ~56 kDa and ~15 kDa and are indicated by green and brown arrows, respectively. The bottom panel shows the results of the MS analysis on the *Tco*PYK protein band. The detected peptides are highlighted in green. MS analysis recovered several peptides covering up to 64.13% of the entire *Tco*PYK sequence. (**d**) A heterologous sandwich ELISA on recombinant *Tco*PYK, *Tbr*PYK, and *Lme*PYK demonstrates that the Nb44/Nb42 pair is specific for *Tco*PYK.

An anti-*T*. *congolense* secretome Nb library was generated by immunising an alpaca with a mixture of the secretomes of *T*. *congolense* strains IL1180 and IL3000 (Supplementary Fig. S1). The panning strategy adopted in this work resulted in the identification of five distinct Nbs. All Nbs were tested for their potential to recognise *T*. *congolense* secretome in a sandwich ELISA. In this set-up, the capturing Nbs are His-tagged (NbH), whereas the detecting Nbs possess an HA-tag (NbHA). Every possible Nb pair was tested on *T*. *congolense* infected mouse serum, leading to 25 combinations. From Fig. 1a it can be seen that two Nb pairs result in a clear signal: Nb42/Nb42 and Nb44/Nb42. As the goal of the work was to develop a heterologous sandwich assay (i.e., capturing and detecting Nb must differ), only the Nb44/Nb42 pair was considered. Interestingly, the roles of these two Nbs in the heterologous sandwich cannot be inverted, as the Nb42/Nb44 combination yields no signal (Fig. 1a).

The specificity of the Nb44/Nb42 pair was verified by testing sera from mice infected with various *Trypanosoma* and one *Leishmania* species. *T*. *congolense* secretome samples and naive mouse sera were employed as positive and negative controls, respectively. Only *T*. *congolense* infections provide positive signals, albeit with varying intensities depending on the strain (Fig. 1b). Infections with other trypanosomatids did not result in any detection.

### The Nb44/Nb42 pair targets *T*. *congolense* pyruvate kinase

Next, the identification of the parasite antigen targeted by the Nb44/Nb42 pair was ensued. To this end, an immuno-capturing experiment was combined with mass spectrometry. SDS-PAGE analysis of an immuno-capturing with Nb42 on *T*. *congolense* IL3000 secretome shows the occurrence of a single protein band with a molecular mass between 55 kDa and 70 kDa in the captured fraction. Mass spectrometry on the excised band identifies the target as *T*. *congolense* pyruvate kinase (Uniprot ID: G0UYF4), a well-known and well-conserved glycolytic enzyme (Fig. 1c).

To verify that the Nb44/Nb42 pair specifically targets *T*. *congolense* pyruvate kinase, the sandwich ELISA was performed with recombinant *T*. *congolense* pyruvate kinase (*Tco*PYK), *T*. *brucei brucei* pyruvate kinase (*Tbr*PYK), and *L*. *mexicana* pyruvate kinase (*Lme*PYK). The production in and purification from *E*. *coli* of these three enzymes is described in Supplementary Fig. S2. As can be seen from Fig. 1d, the Nb44/Nb42 pair only yields a positive signal for *Tco*PYK, thereby confirming the target’s identity and the assay’s specificity.

### Nb42 and Nb44 recognise distinct epitopes on *T*. *congolense* pyruvate kinase

Before further development of the Nb44/Nb42 immunoassay, the interactions of both Nbs with *Tco*PYK were characterised through a combination of surface plasmon resonance (SPR) and analytical size exclusion chromatography (SEC).

**Figure 2:**
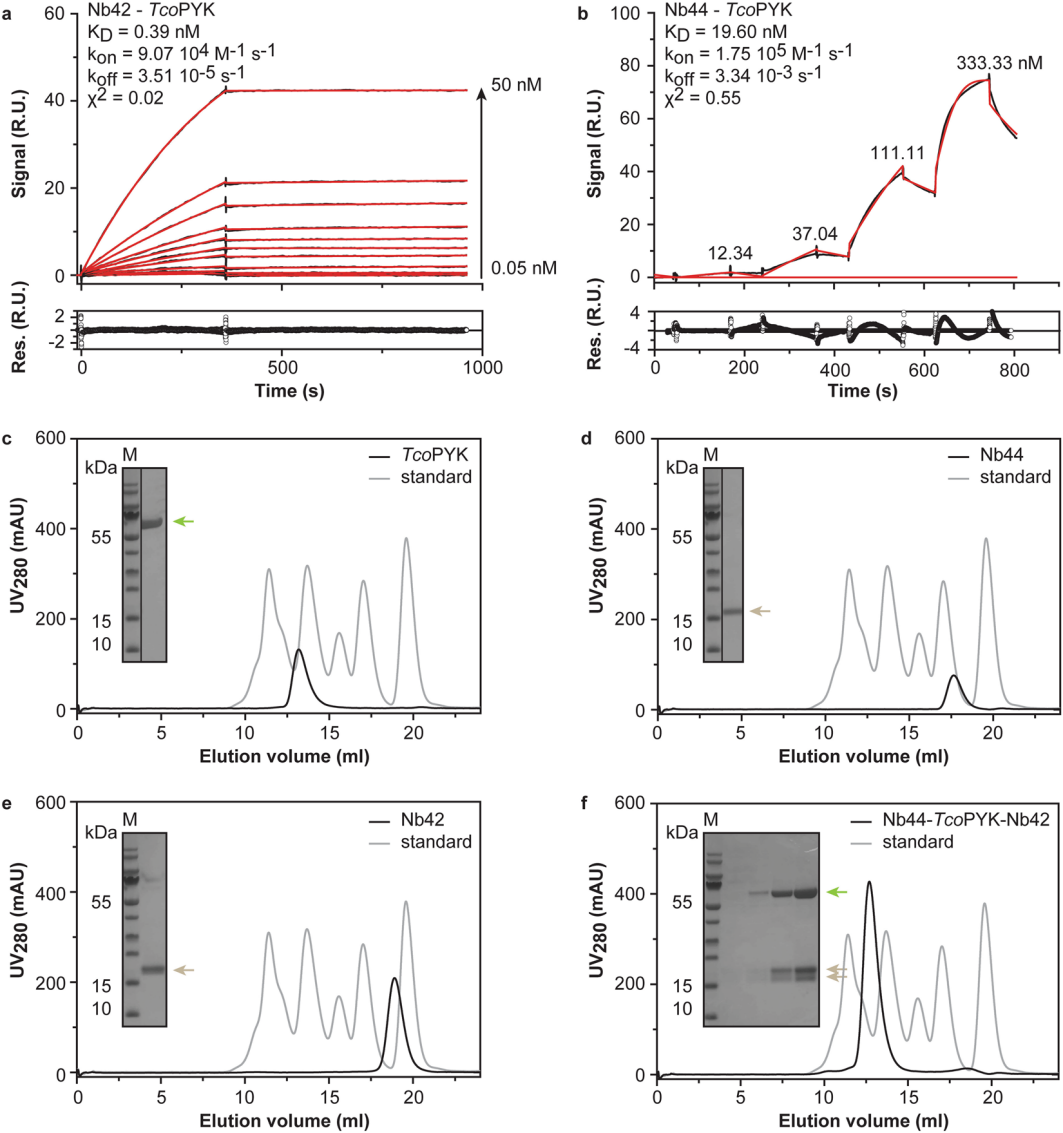
Nb42 and Nb44 recognise distinct epitopes on *T*. *congolense* pyruvate kinase. (**a**,**b**) SPR data recorded for the Nb42-*Tco*PYK (**a**) and Nb44-*Tco*PYK (**b**) interactions with the Nbs as ligands and *Tco*PYK as the analyte. The top panels display the sensorgrams (black traces) and the fit to the data with a 1:1 Langmuir binding model (red traces). The residuals of the fit are shown in the bottom panels. (**c**–**f**) Investigation of the formation of a tripartite (Nb44)_2_: (*Tco*PYK)_4_: (Nb42)_4_ complex via analytical SEC: *Tco*PYK (**c**) Nb44 (**d**) Nb42 (**e**) and Nb42 mixed with the preformed Nb44:*Tco*PYK complex in the correct stoichiometric amounts (**f**). All experiments were performed on an ENRICH 650 10/30 column. The black and grey traces represent the chromatograms of the different protein samples and the BioRAD gel filtration standard, respectively. In all figures, the inset shows an SDS-PAGE analysis of the elution peaks. *Tco*PYK (MM = 56.2 kDa), Nb44 (MM = 14.3 kDa), and Nb42 (MM = 16.4 kDa) are indicated by the green and brown arrows, respectively. Lane M, Prestained Protein Molecular Weight Marker (ThermoFischer Scientific).

The Nb-*Tco*PYK interactions were first investigated by SPR. For the Nb42:*Tco*PYK complex, a data set was collected according to the standard format (Fig. 2a), while for Nb44 data for the interaction with *Tco*PYK were recorded in the format of a kinetic titration^50^ (Fig. 2b) due to the absence of a suitable regeneration condition. Analysis of the SPR data with a 1:1 binding model reveals that the Nb-*Tco*PYK interactions have affinities in the nM range, with Nb42 exhibiting the tightest binding (Fig. 2, panels a and b).

Next, a titration experiment monitored by analytical SEC was carried out to determine the stoichiometries of the Nb42:*Tco*PYK and Nb44:*Tco*PYK complexes. As expected for pyruvate kinases^51^, *Tco*PYK behaves as a tetramer (Supplementary Figs S3 and S4). Interestingly, the Nb42:*Tco*PYK and Nb44:*Tco*PYK complexes display distinct stoichiometries (4:4 and 2:4, respectively). While a single *Tco*PYK tetramer can bind 2 Nb44 molecules, 4 binding sites are available for Nb42 (Supplementary Figs S3 and S4). This suggests that Nb44 and Nb42 recognise distinct, non-competing epitopes on *Tco*PYK. To verify this hypothesis, analytical SEC was employed to assess whether a tripartite (Nb44)_2_: (*Tco*PYK)_4_: (Nb42)_4_ complex could be formed. Because of the unidirectional character of *Tco*PYK binding by the Nbs, Nb44 was allowed to interact with *Tco*PYK prior to Nb42 during sample preparation. The results demonstrate that the tripartite (Nb44)_2_: (*Tco*PYK)_4_: (Nb42)_4_ complex elutes as a single peak containing all three proteins indicating that Nb44 and Nb42 do not compete for the same *Tco*PYK epitope (Fig. 2, panels c through f). These findings provide evidence that both Nbs bind distinct epitopes on *Tco*PYK, thereby advocating their further use in the development of a heterologous sandwich ELISA and LFA.

### Optimisation of the Nb44/Nb42 ELISA assay buffer counteracts the plasma matrix effect

**Figure 3:**
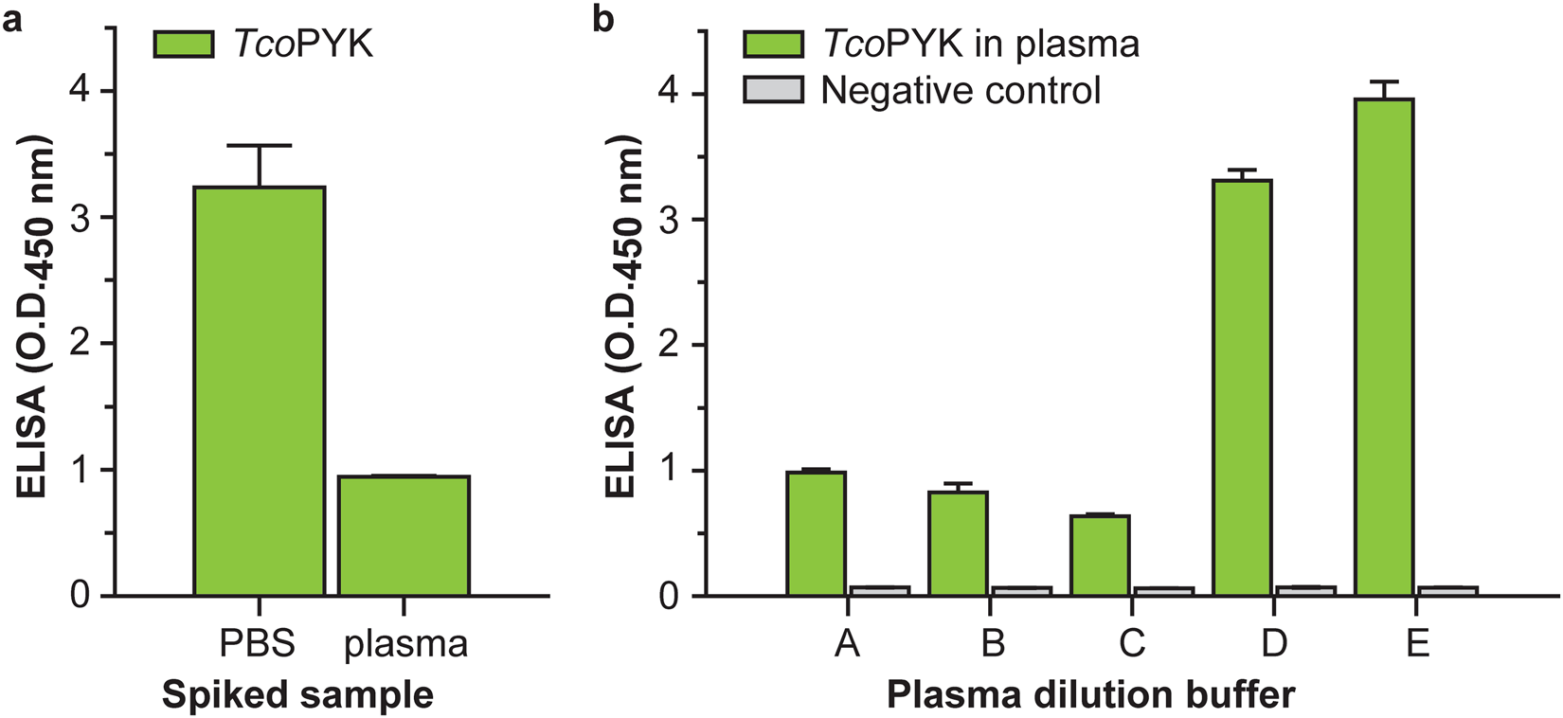
Optimisation of the Nb44/Nb42 ELISA assay buffer counteracts the plasma matrix effect. (**a**) Nb44/Nb42 ELISA tested on PBS and naive mouse plasma spiked with *Tco*PYK at a concentration of 1 *μ*g ml^−1^. (**b**) Nb44/Nb42 ELISA tested on naive mouse plasma spiked with *Tco*PYK at a concentration of 1 *μ*g ml^−1^ and diluted three-fold in various assay buffers (A: PBS, B: PBS + 100 mM NaCl, C: PBS + 300 mM NaCl, D: PBS + 0.5% Tween20, E: PBS + 1.0% Tween20).

To determine those Nb44 and Nb42 amounts yielding the highest ELISA signal, a checkerboard format was employed and tested on PBS spiked with *Tco*PYK. As can be seen from Supplementary Fig. S5, the amount of Nb44 has the largest influence on the ELISA’s read-out. Upon testing the Nb44/Nb42 ELISA on naive mouse serum spiked with *Tco*PYK, a so-called ‘plasma matrix effect’ was observed. A ‘plasma matrix effect’ is defined as “*the direct or indirect alteration or interference in response due to the presence of unintended analytes* (*for analysis*) *or other interfering substances in the sample*”^52,53^. Indeed, from Fig. 3a it is clear that the sensitivity of the assay drops significantly when the query sample consists of naive mouse serum spiked with *Tco*PYK. In an attempt to overcome this effect, the spiked mouse serum was diluted in various buffers and subsequently evaluated in the Nb44/Nb42 immunoassay. The best results are obtained when the spiked serum samples are diluted 3-fold in PBS containing 1% Tween20, indicating that the inclusion of a non-ionic detergent in the reaction mixture alleviates the plasma matrix effect (Fig. 3b).

### The versatility of Nbs can be exploited to improve the performance of the Nb42/Nb44 ELISA

**Figure 4:**
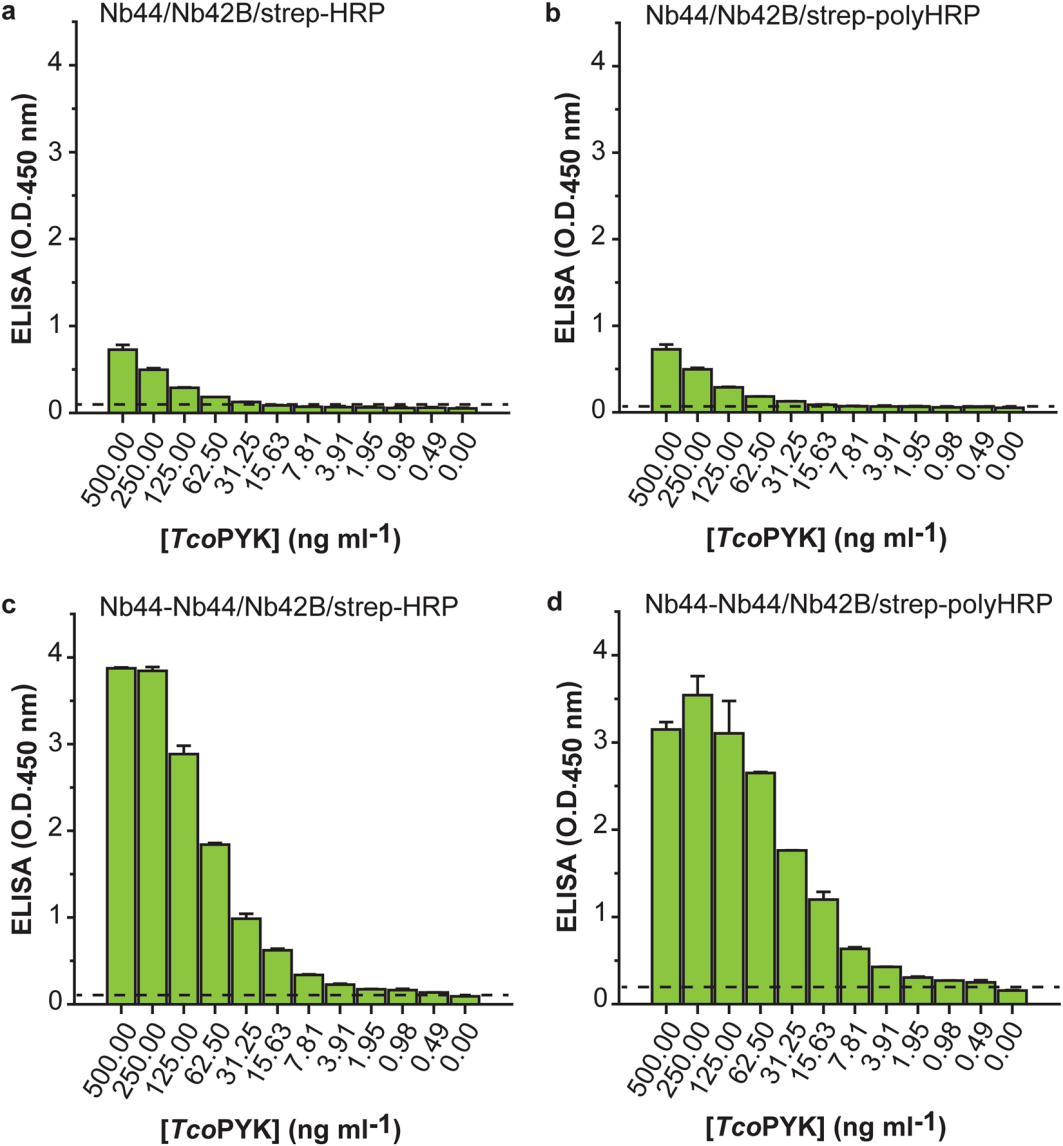
The versatility of Nbs can be exploited to improve the performance of the Nb42/Nb44 ELISA. Nb44/Nb42 ELISA tested on naive mouse serum spiked with a dilution series of *Tco*PYK using monovalent Nb44H for capturing, biotinylated Nb42B for detection, and strep-HRP for assay development (**a**) monovalent Nb44H for capturing, biotinylated Nb42B for detection, and strep-polyHRP for assay development (**b**) bivalent Nb44-Nb44H for capturing, biotinylated Nb42B for detection, and strep-HRP for assay development (**c**) and bivalent Nb44-Nb44H for capturing, biotinylated Nb42B for detection, and strep-polyHRP for assay development (**d**). The dashed line displays the ELISA LoD (LoD = mean_blank_ + 3.3 * SD_blank_). The concentration of *Tco*PYK represents the concentration prior to 3-fold dilution into the assay buffer.

Further optimisation of the Nb44/Nb42 ELISA consisted of reducing the number of assay steps (and hence, assay time). This was achieved by generating a biotinylated version of Nb42 (Nb42B), which can directly be detected via a streptavidin-enzyme conjugate (streptavidin-horse radish peroxidase; strep-HRP) thereby omitting one assay step compared to the set-up with Nb42HA (which entails an additional incubation with biotinlyated anti-HA mouse monoclonal). When tested on naive mouse serum spiked with *Tco*PYK, the Nb44/Nb42B/strep-HRP combination yields a limit of detection (LoD) of around 16 ng ml^−1^ (Fig. 4a). Next, to amplify the ELISA signal upon assay development, strep-HRP was replaced by strep-polyHRP, which constitutes of streptavidin conjugated with polymers of HRP. This modification to the protocol enhanced the LoD to 2 ng ml^−1^ (Fig. 4b). Finally, Nb44 was tailored in an attempt to improve the ELISA’s LoD. From Supplementary Fig. S5 it is clear that the amount of Nb44 limits the assay’s sensitivity. Given that Nb44 has a lower affinity for *Tco*PYK compared to Nb42 as determined via SPR (Fig. 2), it was reasoned that employing a bivalent version of Nb44 (Nb44-Nb44) as a capturing agent would increase the sensitivity of the assay. Indeed, a comparison between the Nb44/Nb42 and Nb44-Nb44/Nb42 formats reveals that the use of Nb44-Nb44 as a capturing agent markedly improves the LoD to ~0.5 ng ml^−1^ (Fig. 4, panels c and d).

In conclusion, the versatility of Nbs allowed a 32-fold enhancement of the ELISAs LoD. The final format employs bivalent Nb44-Nb44H as a capturing agent, monovalent Nb42B for detection, and strep-polyHRP for assay development. This Nb44-Nb44/Nb42 ELISA was evaluated as such in mouse infection trials (see below).

### The Nb44/Nb42 pair forms the basis for a *T*. *congolense* specific LFA

**Figure 5:**
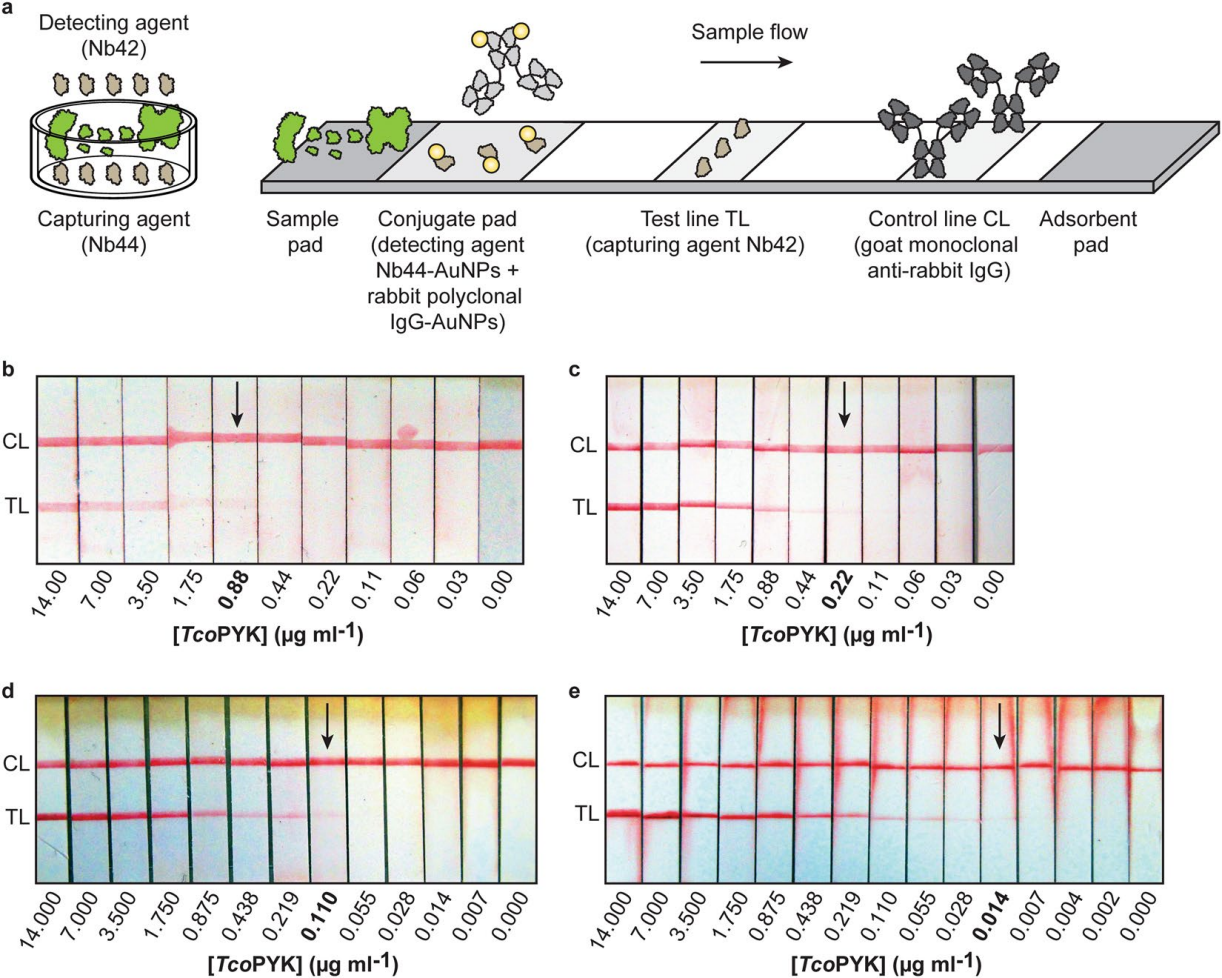
Generation of a Nb-based LFA for detection of *T*. *congolense*. (**a**) Schematic representation of the Nb44/Nb42 sandwich ELISA (left panel) and Nb44/Nb42 LFA (right panel). In both panels, the *T*. *congolense* antigens are coloured in green, while the Nbs are shown in brown. IgGs are displayed in light (rabbit polyclonal) and dark (goat monoclonal) grey. The AuNPs are shown as yellow circles and are not drawn to scale with the Nbs and IgGs. (**b**–**e**) LFA read-outs for (**b**) PBS spiked with a dilution series of *Tco*PYK using direct coating of Nb42 on the TL, (**c**) PBS spiked with a dilution series of *Tco*PYK using streptavidin-based coating of biotinylated Nb42 on the TL, (**d**) naive mouse serum spiked with a dilution series of *Tco*PYK (streptavidin-based coating of biotinylated Nb42 on the TL) and (**e**) naive mouse serum spiked with a dilution series of *Tco*PYK and diluted three-fold in PBS containing 1% methylcellulose and 1% Tween20 (streptavidin-based coating of biotinylated Nb42 on the TL). The visual LoD was established based on the last *Tco*PYK concentration at which a TL band could be observed (indicated by the black arrow). 50 *μ*l of sample was spotted onto the sample pad.

Since Nb42 and Nb44 recognise distinct *Tco*PYK epitopes, the two Nbs were employed to develop a *T*. *congolense* specific LFA. Because of the unidirectional character of target antigen binding by the Nb44/Nb42 pair, the capturing and detecting roles of the two Nbs have to be inverted compared to the sandwich ELISA set-up. While in a sandwich ELISA the capturing agent interacts with the target antigen prior to the detecting agent, this is no longer true in an LFA (*i*.*e*. the detecting agent interacts with the target antigen before the capturing agent). Thus, in the LFA format, Nb44 and Nb42 act as detecting and capturing agents, respectively, instead of *vice versa* as for the sandwich ELISA (Fig. 5a). The LFA control line consists of goat monoclonal anti-rabbit IgG which recognises rabbit polyclonal IgG AuNPs that are also applied to the conjugate pad with the Nb44-AuNPs since a monoclonal anti-Nb is currently not available.

The Nb44/Nb42 LFA effectively detects *Tco*PYK in PBS with a visual LoD of ~880 ng ml^−1^ (Fig. 5b). The sensitivity could further be increased by immobilising Nb42B on a streptavidin-coated test line (Fig. 5c). The LFA was also tested on plasma samples from naive mice spiked with various amounts of *Tco*PYK. The initial visual LoD (Fig. 5d) was further improved by optimising the conditions under which the Nbs and antigen are allowed to interact. This was performed by taking two parameters into account: (i) the sample viscosity, which influences the sample flow speed and thus contact time between the Nbs and *Tco*PYK, and (ii) the plasma matrix effect, which can have a negative impact on antigen detection as described earlier for the heterologous sandwich ELISA. An assay buffer was designed to simultaneously reduce both the sample flow speed and plasma matrix effects. The final assay buffer contains 1% methylcellulose (a component that increases the sample’s viscosity) and 1% Tween20 (which deals with the matrix effect as observed previously). As can be seen from Fig. 5e, a significant improvement in detection is obtained when naive mouse plasma spiked with *Tco*PYK is diluted three-fold in the assay buffer containing both components (visual LoD of around 14 ng ml^−1^).

The final LFA format employs Nb44-AuNPs for detection, Nb42B on a streptavidin-coated test line as capturing agent, and requires a 3-fold dilution of the query sample in PBS containing 1% methylcellulose and 1% Tween20. This Nb44/Nb42 LFA was evaluated as such in mouse and cattle infection trials (see below).

### The Nb44-Nb44/Nb42 ELISA and Nb44/Nb42 LFA can be used as test-of-cure tools during experimental *T*. *congolense* infections in mice

**Figure 6:**
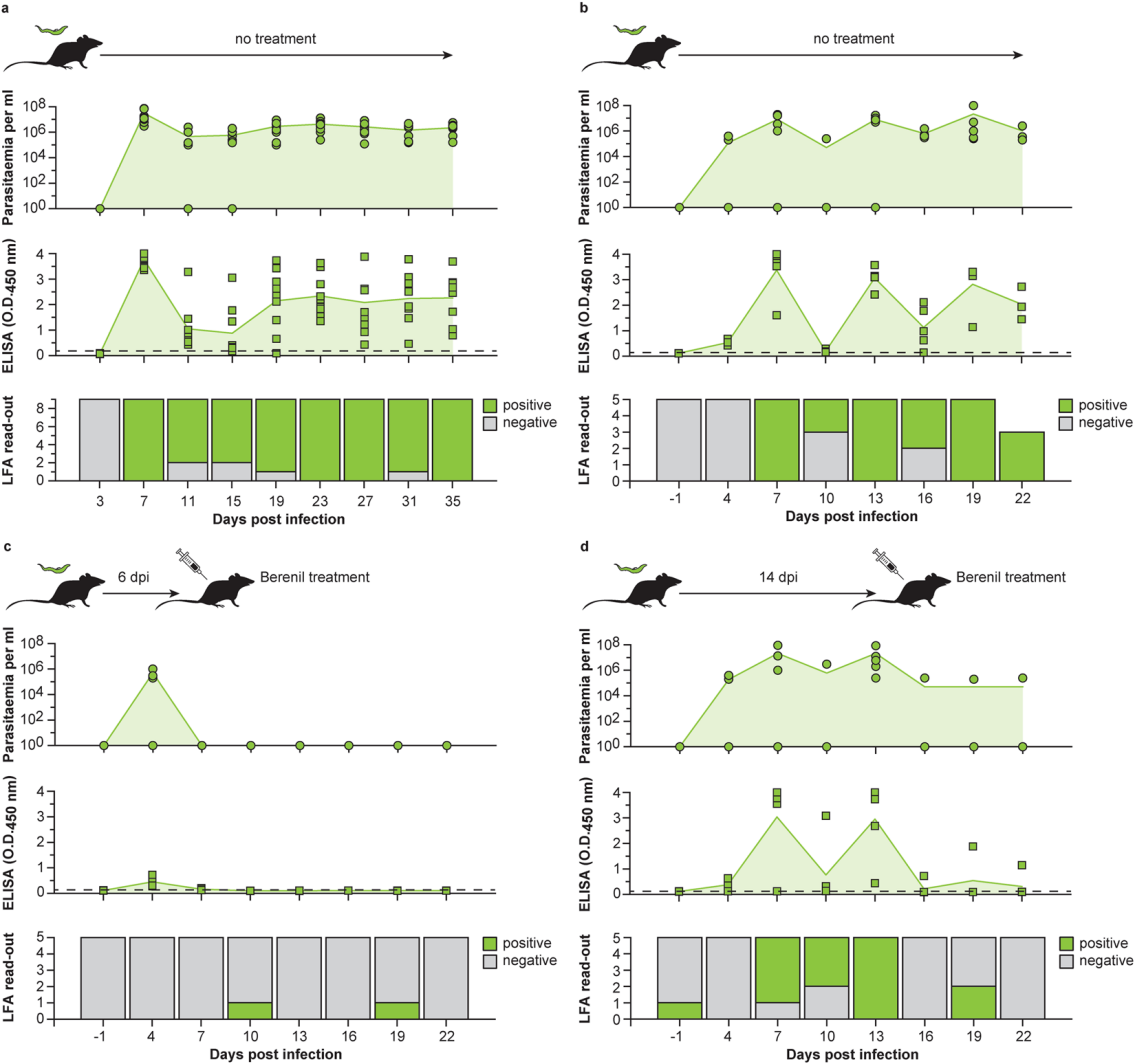
The Nb44-Nb44/Nb42 ELISA and Nb44/Nb42 LFA can be used as test-of-cure tools during experimental *T*. *congolense* infections in mice. (**a**) The detection of target antigen via the Nb44-Nb44/Nb42 ELISA and Nb44/Nb42 LFA correlate well with parasitaemia. The top panel displays the parasitaemia as determined by microscopy monitored over the course of the infection in C57BL6/C mice infected with *T*. *congolense* Tc13 (n = 9). The middle panel shows the antigenaemia observed by the Nb44-Nb44/Nb42 ELISA. The mean values for parasitaemia and antigenaemia are represented by the green line with light green fill. The dashed line displays the ELISA detection cut-off value. The bottom panel shows the results of the LFA read-outs on the infected mice. (**b–d**) C57Bl6/C mice infected with *T*. *congolense* Tc13 were divided into three groups (Group 1 (n = 5), left untreated; Group 2 (n = 5), treated with Berenil at 6 dpi; Group 3 (n = 5), treated with Berenil at 14 dpi) and antigenaemia and parasitaemia were followed over time. The results for Group 1, Group 2, and Group 3 are shown in panels (b–d), respectively. The panels and colour codes are the same as for panel (a).

Both Nb-based assays were evaluated for their potential to differentiate between ongoing and past infections in an experimental mouse model. In a first step, the correlation between antigenaemia and parasitaemia was determined by examining blood samples taken from C57BL6/C mice (n = 9) infected with *T*. *congolense* Tc13 at various time points using the Nb44-Nb44/Nb42 ELISA and Nb44/Nb42 LFA (antigenaemia) and microscopy (parasitaemia). The ELISA and LFA scored positive for most infected mice and correlated relatively well with parasitaemia during the course of the infection (Fig. 6a). The positive score rates for LFA and microscopy were similar (although both did not always score negative on the same samples), while the positive score rate for ELISA was higher through-out the entire follow-up (Table 1).

**Table 1:** Kappa coefficient analysis of the correlation between the employed diagnostic methods (microscopy, ELISA, LFA).

Microscopy			Microscopy			LFA		
ELISA	(−)	(+)	LFA	(−)	(+)	ELISA	(−)	(+)
(−)	9	1	(−)	10	5	(−)	10	0
(+)	4	65	(+)	3	61	(+)	5	64
**Kappa ± 95% CI** 0.746 ± 0.211			**Kappa ± 95% CI** 0.653 ± 0.221			**Kappa ± 95% CI** 0.764 ± 0.195		

In a ‘test-of-cure’ experiment, mice infected with *T*. *congolense* Tc13 were divided into three groups and antigenaemia and parasitaemia were followed over time. Group 1 (n = 5) was left untreated, while Groups 2 and 3 (both n = 5) were treated with Berenil at 6 and 14 days post-infection, respectively. As expected Group 1 samples display a similar behaviour as observed previously (Fig. 6b). After Berenil drug treatment of the infected mice (Groups 2 and 3), no *Tco*PYK could be detected in most cases suggesting that the target antigen disappears from the blood stream as soon as 24 hours post-treatment, coinciding with parasite clearance (Fig. 6, panels c and d). Only a single mouse in Group 3 had persistent parasitaemia, which could also be detected by the Nb44-Nb44/Nb42 ELISA.

### The Nb44/Nb42 LFA can be employed to detect experimental *T*. *congolense* infections in cattle

The diagnostic performance of the Nb44/Nb42 LFA was examined by testing stored plasma samples from naive Holstein cows (n = 15) and Holstein cows experimentally infected with *T*. *vivax* STIB 719 (n = 22) and *T*. *congolense* KONT 2/133 (n = 24). Based on the collected data, the LFA’s preliminary sensitivity and specificity, positive predictive value (PPV), and negative predictive value (NPV) were calculated (Table 2).

**Table 2:** Preliminary evaluation of sensitivity, specificity, positive and negative predicted values for the Nb44/Nb42 LFA performed on plasma samples from experimentally infected cattle.

	Cattle sera infected with		
	*T*. *congolense*	Naive	*T*. *vivax*
Positive	19	1	2
Negative	5	14	20
Total	24	15	22
Sensitivity ± 95% CI (%)	79.17 (19/24) ± 16.25		
Specificity ± 95% CI (%)	91.89 (34/37) ± 8.80		
PPV ± 95% CI (%)	86.36 (19/22) ± 14.34		
NPV ± 95% CI (%)	87.18 (34/39) ± 10.47		

The samples were obtained from a stored GALVmed plasma sample collection at Clinvet, South Africa. The collection contains plasma samples of Holstein cows that were experimentally infected with *T*. *vivax* STIB 719 or *T*. *congolense* KONT 2/133. Because these animals were involved in drug development programs, they were treated as soon mild disease symptoms developed combined with an observed hematocrit value lower than 25%.

**Figure 7:**
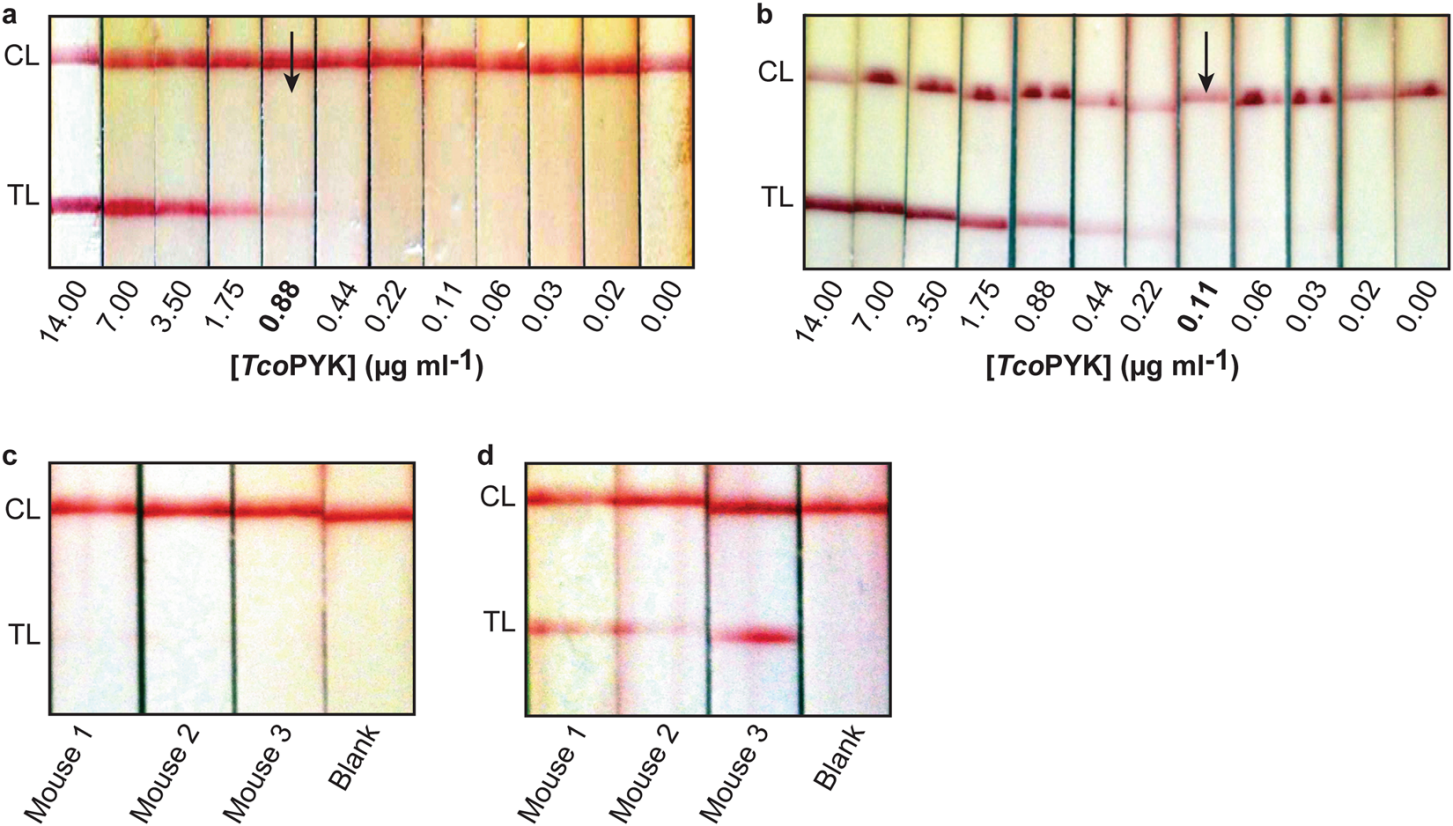
The Nb44/Nb42 LFA can be employed to detect experimental *T*. *congolense* infections in cattle. (**a**,**b**) LFA read-outs for naive cattle serum spiked with a dilution series of *Tco*PYK using monovalent Nb44-AuNPs (**a**) and bivalent Nb44-Nb44-AuNPs (**b**). In both cases, the samples were diluted three-fold in PBS containing 1% methylcellulose and 1% Tween20. (**c**,**d**) LFA read-outs for sera from mice infected with *T*. *congolense* using monovalent Nb44-AuNPs (**c**) and bivalent Nb44-Nb44-AuNPs (**d**). In both cases, the samples were diluted three-fold in PBS containing 1% methylcellulose and 1% Tween20. In all LFAs, the test line consists of a streptavidin-based coating of biotinylated Nb42. The visual LoD was established based on the last *Tco*PYK concentration at which a TL band could be observed (indicated by the black arrow). 50 *μ*l of sample was spotted onto the sample pad.

In an effort to further optimise the LFA’s performance on cattle samples, its visual LoD was examined on naive bovine plasma spiked with *Tco*PYK. Compared to mice plasma samples (Fig. 5e), the visual LoD in this case was inferior and differed ~60-fold (Fig. 7a). The observed reduced performance of the LFA is probably due to plasma matrix effects as it has been documented that the composition of plasma differs notably between animal species^54^. The LFA’s visual LoD on bovine plasma samples spiked with *Tco*PYK was improved 8-fold by employing bivalent Nb44-Nb44-AuNPs as a detecting agent (Fig. 7b). To demonstrate that the use of bivalent Nb44-Nb44-AuNPs would also increase the LFA’s sensitivity in an infection setting, sera of *T*. *congolense* infected mice that previously yielded a negative LFA result but a positive ELISA signal (Fig. 6, days 11 and 15 post-infection) were re-evaluated using the optimised LFA. Indeed, the visual LoD of the LFA is improved as clear bands are now visible for these plasma samples (Fig. 7, panels c and d).

## Discussion

A large contributor for the failed eradication of many infectious diseases in developing countries is poor consultation and a lack of access to diagnostic equipment. RDTs possess the potential to overcome this diagnostic gap in resource-limited areas^55^. However, RDT development for tropical diseases, such as African trypanosomosis, has encountered challenges of technical, infrastructural, and socio-economic nature^56^. One of the technical barriers early on in the development of an antigen-based RDT is the inadequate characterisation of the antigen, the detecting and capturing antibodies, and the antigen-antibody interactions. An ideal biomarker should be specific, abundant, stable, quantitatively meaningful, universally present in hosts, accessible in the sample fluids, timely observable and well characterised^57^. Proteins present in a parasite’s secretome may fulfill the aforementioned requirements. The secretome represents the proteome fraction that is actively secreted by the parasite. However, it should be noted that some proteins found in the secretome may be present as a consequence of parasite death and lysis. The *T*. *congolense* secretome employed for Nb generation in this work was produced via the method described by Holzmuller and colleagues, which ensures a parasite viability of over 97% during secretome preparation^58^.

In this paper, we describe the identification of *Tco*PYK as a biomarker for *T*. *congolense* infections through the generation of an unbiased Nb library against the parasite’s secretome. The premise of the approach is straightforward: (i) a camelid is immunised with a parasite’s secretome, (ii) (a pair of) parasite-specific Nbs are identified and iii) subsequently employed to unravel the identity of the targeted antigen. Several advantages can be linked to this strategy. First, the Nb library is generated against native antigen(s), which enables the selected binders to recognize the target protein(s) with their naturally occurring post-translational modifications (if any are present). Second, using a protein fraction both during immunisation and phage display skews the affinity maturation and *in vitro* selection of Nbs towards those proteins that are the most abundant and/or immunogenic. This increases the likelihood that eventually enough Nbs can be generated for assay development and that these Nbs detect antigen(s) that are present in sufficient quantities *in vivo*. This rationale has been applied previously using *T*. *congolense* soluble proteome and has resulted in the development of a *T*. *congolense* specific homologous sandwich ELISA targeting glycosomal fructose-1,6-bisphosphate aldolase (*Tco*ALD)^42^. However, because of the homologous nature of this assay, it was more challenging to translate the sandwich ELISA into an LFA. Hence, the endeavour towards developing a Nb-based LFA for *T*. *congolense* diagnosis was pursued in this work. The anti-*T*. *congolense* secretome Nb library yielded a Nb pair (Nb42 and Nb44) that specifically targets the glycolytic enzyme *T*. *congolense* pyruvate kinase (*Tco*PYK).

It is intriguing that yet another glycolytic enzyme is identified as a diagnostic biomarker. Other examples are provided by (i) *Plasmodium vivax* aldolase for malaria^59^, (ii) human pyruvate kinase, *α*-enolase, and triose phosphate isomerase for apoptosis^60^, (iii) and human muscle pyruvate kinase isoform 2 (PKM2) in colorectal cancer^61^. Interestingly, a significant number of reports in the recent literature describe additional non-glycolytic functions of glycolytic enzymes^62–66^. In the case of parasites, moonlighting glycolytic enzymes have been reported to be exposed as surface antigens^67–70^ or belong to the parasite’s secretome, which constitutes the fraction of parasite proteins released into the extracellular environment in so-called ‘extracellular vesicles (EVs)’^71,72^. Both *Tco*ALD and *Tco*PYK have been shown to be part of the parasite’s secretome, which has been described for both animal and human trypanosomes^58,73–75^. In the case of trypanosomes, EVs are thought to play an important role in the host-parasite interaction^75^. How the individual secretome members affect the host-pathogen relationship remains enigmatic.

The observations in past reports^42,59^ and this work demonstrate that glycolytic enzymes can be employed as specific biomarkers to distinguish between species within a given parasite genus. This is surprising given the relatively high degree of sequence conservation for glycolytic enzymes. For the *T*. *congolense* biomarkers identified by our group (*Tco*ALD^42^ and *Tco*PYK identified in this work) the sequence identities with *Tbr*ALD and *Tbr*PYK are 94.1% and 88.0%, respectively. For the Nb-based *T*. *congolense* immunoassay targeting *Tco*ALD^42^, we have recently shown that the molecular basis for specificity lies in the assay’s homologous sandwich design^76^. In the case of the Nb44/Nb42 assays, the reasons for their high *T*. *congolense* specificity remain unclear. The characterisation of the Nb-*Tco*PYK interactions performed in this work reveals two features of the Nb44/Nb42 assays. First, the Nb pair only works in a unidirectional manner. When Nb44 and Nb42 are employed as capturing and detecting Nbs, respectively, both the ELISA and LFA display a clear signal. However, when the roles of both Nbs are swapped, no signal is observed. Second, a tripartite (Nb44)_2_: (*Tco*PYK)_4_: (Nb42)_4_ complex can be generated, suggesting that both Nbs bind distinct, non-competing epitopes. In the absence of further detailed studies of these interactions, the molecular basis for the assay’s specificity and unidirectionality remains yet unknown.

The Nb44/Nb42 pair was first tested in the format of a heterologous sandwich ELISA. In order to limit the number of assay steps, Nb42HA was replaced by its biotinylated counterpart Nb42B. The LoD of the Nb44/Nb42 ELISA (using Nb42B and strep-HRP) could be improved by a factor 32 by optimising the valency of the capturing agent Nb44 and the properties of the streptavidin-enzyme conjugate used for assay development. These modifications highlight the versatility of Nbs as detection tools. Because of their small size, they can easily be incorporated into multivalent and/or tagged constructs^48^ to overcome significant limitations. In this case, the gain in sensitivity obtained via the bivalent Nb44-Nb44 construct is probably due to an increased apparent affinity of the Nb-antigen interaction through avidity. In an experimental mouse model, the optimised heterologous Nb44-Nb44/Nb42 ELISA detects target antigen as soon as 4 days post-infection. More importantly, *Tco*PYK could not be detected in animals that were cleared of infection through Berenil treatment (clearance as soon as 24 h post-treatment occurred in most of the mice). Hence, it seems that living parasites are required in order for the target antigen to be detected by the Nb44-Nb44/Nb42 ELISA. It should be noted that, throughout the experiments, the absence of a linear correlation between the antigen levels in blood and the parasitaemia was observed (*i*.*e*. high levels of parasitaemia do not necessarily correspond to high OD values in ELISA). This is due to a multitude of uncertainties (and/or untraceable events): (i) containment of antigen in the blood stream (present in EVs and/or freely circulating protein in blood; ratio between free and vesicular antigen remains unknown) (ii) fitness of the parasite and amount of antigen released; (iii) likely differential clearance from blood of free antigen and vesicular antigen; (iv) involvement of the host immune system in clearing the antigen (e.g. via host antibodies against *Tco*PYK and/or effect of inflammation on clearance); (v) the antigen dynamics: the balance between antigen release in the blood and its clearance rate via multiple pathways (hepatic versus renal clearance), *etc*. Nonetheless, the above-mentioned results suggest that the Nb44-Nb44/Nb42 ELISA may serve as a test-of-cure application during drug screening programs or in follow-up field studies after treatment with a commercial drug. Additionally, given that the Nb44-Nb44/Nb42 ELISA is highly specific for *T*. *congolense*, it could be a valuable tool to accurately determine the prevalence and incidence of *T*. *congolense* infections in epidemiology studies.

While the ELISA can be used in a standard laboratory environment, a complementary RDT is necessary for faster decision making in rural areas. The LFA format is a highly suitable one for use under field conditions when it complies with the ASSURED criteria^19^. While procedures to couple conventional antibodies to AuNPs for a visual read-out are well established, manufacturing Nb-AuNP conjugates is less straightforward. Recently, we demonstrated that more acidic Nbs possess the right charge balance to generate stable Nb-AuNP conjugates through physical adsorption^49^. Since Nb42 and Nb44 (i) are relatively acidic (pI values of 6.42 and 5.55, respectively), and (ii) bind distinct *Tco*PYK epitopes, the heterologous sandwich ELISA could be translated into an LFA. The unidirectionality of the Nb44/Nb42 pair imposes the roles of the Nbs in the LFA format. Nb44 should be used as the detection agent and to generate stable Nb-AuNPs, which is convenient since it is the most acidic Nb^49^. Nb42 should be used as the capturing agent and immobilised on the test line. A streptavidin-based immobilisation method (also preferred by others^41^) was chosen for this purpose as this improves the analytical sensitivity. This is probably due to a better orientation of the capturing Nb which improves antigen recognition and an increased immobilisation efficiency on the nitrocellulose membrane since it is known some small proteins do not adhere easily onto the surface material^77^. The detection label, pads and membranes constituting the LFA strips were standard as more complicated material, design and alternative labelling strategies would raise manufacturing costs and diminish speed and user-friendliness^78^. The Nb44/Nb42 LFA was first tested and optimised on mice sera spiked with *Tco*PYK and subsequently tested in the mouse infection trials. Compared to the Nb44/Nb42 ELISA, the LFA seems to possess a higher false negative rate during early infection stages, when parasites are barely present in the host’s blood. Most likely this is due to a technical limitation of the LFA format as a detection system rather than a failure of the Nbs to capture low quantities of analyte in *T*. *congolense* infected blood. The Nb44/Nb42 ELISA, which is based on the same Nb pair as the LFA, might display a higher sensitivity because of longer incubation times and an enzyme-mediated signal amplification step.

Finally, the LFA was also tested on serum samples from cattle experimentally infected with *T*. *congolense*. This initial study shows that the Nb44/Nb42 LFA in its current format could be useful for cattle screening at the herd level. In addition, given the high specificity of the test (92%), a positive score would allow for a reliable decision making in anti-trypanosomosis treatment. As future perspective, the LFA described here could be amenable to further optimisation to allow its use for decision making based on single individual cattle screenings. Our first efforts to increase the LFA’s sensitivity in cattle samples entails employing AuNPs coated with the bivalent Nb44-Nb44 construct. In future work, this could be improved even further by optimizing the composition of the assay buffer to overcome the ‘plasma matrix effect’ associated with cattle sera^54^. In conclusion, we have paved the road towards developing the first Nb-based LFA for the specific diagnosis of active *T. congolense* infections via detection of a parasite antigen (pyruvate kinase). After further development, the Nb44/Nb42 LFA will be evaluated in a large field study to assess the assay’s true performance (NPV and PPV) as an RDT in a realistic setting.

## Methods

### Ethical statement

All animal experiments were carried out according to directive 2010/63/EU of the European parliament for the protection of animals used for scientific purposes and approved by the Ethical Committee for Animal Experiments of the Vrije Universiteit Brussel (clearance numbers 14-220-8 and 14-220-9).

Bovine serum/plasma samples used in this study were kindly provided by Galvmed and tested in Clinvet research innovation (Bloemfontein, South Africa). 62 samples from experimentally infected Holstein cows were analysed using the LFA. These samples include pre-infected samples (n = 15), *T*. *vivax* STIB 719 infected samples (n = 22) and *T*. *congolense* KONT2/133 infected samples (n = 25).

### Nb library construction and phage display onto *T*. *congolense* secretome

Secretomes from two different *T*. *congolense* strains (IL3000 and IL1180) were kindly provided by Dr. Philippe Holzmuller (CIRAD Montpellier, France). Secretomes were prepared as previously described^73^. The details of alpaca immunisation, Nb library generation and panning, Nb cloning, production, and purification are given in the Supplementary Information file accompanying this article.

### Antigen identification by LC-MS

The target antigen recognised by the Nb44/Nb42 sandwich was isolated through an immuno-capturing experiment. Five hundred g *T*. *congolense* IL3000 secretome was mixed with 25 *μ*g Nb42H in a final volume of 300 *μ*l PBS. The mixture was incubated for 2 h at 22 °C. The Nb-antigen complex was isolated using the QuickPick™ IMAC kit (Bio-Nobile) following the kit instructions provided by the manufacturer. Aliquots of 20 *μ*l from each purification step were collected and resolved under reducing conditions on SDS-PAGE using 10% precast polyacrylamide gels (NuPAGE Bis-Tris gels, Thermo scientific), followed by Coomassie-blue staining. The band of interest was excised from the SDS-PAGE gel and analysed by LC-MS/MS spectrometry as described^42^.

### Production and purification of *Tco*PYK, *Tbr*PYK and *Lme*PYK

The genes encoding pyruvate kinase of *T*. *congolense* (*Tco*PYK, Genbank NCBI ID: CCC94421), *T*. *brucei brucei* (*Tbr*PYK, Genbank NCBI ID: X57950) and *L*. *mexicana* (*Lme*PYK, Genbank NCBI ID: X74944) were codon optimised for expression in *E*. *coli*. Gene synthesis and cloning were performed by GenScript. Briefly, the synthesised genes were cloned into pET21b vector (Novagen) using the *Nde*I and *Xho*I restriction sites. All genes were designed such to equip the resulting protein products with a C-terminal protease cleavage site (TEV) and a His-tag. The three constructs were transformed into *E*. *coli* BL21(DE3) cells using the electroporation method. Transformed cells were plated on LB agar plates supplemented with 2% glucose and 100 *μ*g ml^−1^ ampicillin. The details for protein production and purification are given in the Supplementary Information file accompanying this article.

### Surface plasmon resonance

Surface plasmon resonance (SPR) experiments were performed on a BIAcore T200 system (GE Healthcare). A detailed explanation of the experimental set-up is provided in the Supplementary Information file accompanying this article.

### ELISA

Depending on the goal of the experiment, the plates were coated with different capturing material (see different ELISA variants described in the Supplementary Information file accompanying this article). The coated plate was incubated overnight at 4 °C and the excess of non-coated material was removed by washing the plate three times with PBS containing 0.01% Tween20 (PBS-T). Next, blocking of residual protein binding sites was performed by adding 300 *μ*l blocking buffer (5% milk powder in PBS) to each well and the plate was kept for 2 h at room temperature. Subsequently, the plate was washed three times with PBS-T, after which samples were allowed to interact with the capturing material. After incubation for 1 h at room temperature, the plates were subsequently washed three times with PBS-T. Then, 100 *μ*l primary detection reagent was added to the plates at the appropriate concentration. The plates were incubated for 1 h at room temperature and subsequently washed 5 times with PBS-T. The secondary antibody, 100 *μ*l of streptavidin-HRP conjugate (Jackson ImmunoResearch laboratories; diluted to a concentration of 0.5 *μ*g ml^−1^ in protein-free blocking buffer (ThermoFischer Scientific)), was then added to the plate, followed by incubation for 1 h at room temperature. After a final washing step (5 times with PBS-T), the ELISAs were developed by addition of 100 *μ*l of 3,3′,5,5′-tetramethylbenzine (TMB) substrate and subsequent incubation for 25 min at room temperature. The enzymatic reaction was stopped by adding 50 *μ*l 1 M H_2_SO_4_ to the reaction mixture. The plates were read at OD_450*nm*_ with a VersaMax ELISA Microplate Reader (Molecular Devices).

### *Tco*PYK lateral flow assay manufacturing

The LFA gold pad is developed from polyester (VL98 from Shanghai Kinbio Tech. Co., Ltd. Shanghai, China). The nitrocellulose membrane has a capillary flow of 135 s 4 cm^−1^ (HF135, EMD-Millipore). The sample pad consists of glass fibre (Shanghai Kinbio Tech. Co., Ltd. Shanghai, China). Gold nanoparticles were prepared via citrate reduction of hydrogen tetrachloroaurate according to according to established protocols^79,80^. 66 *μ*M of Nb44 or Nb44-Nb44 was adsorbed for 30 min onto 100 ml of pH adjusted gold particles (using 0.2 M K_2_CO_3_). Next, the particles were blocked 20 min with 1% BSA (MilliQ). The particles were concentrated 20x through centrifugation (17.000 g, 30 min.), sprayed onto pre-treated gold pad membranes (4 *μ*l cm^−1^) with the Biodot XYZ3060 dispense platform and dried overnight at 37 °C. Meanwhile a test line was applied with the Biodot dispenser onto a nitrocellulose membrane using a 2:1 molar ratio of Nb42B (1 mg ml^−1^) and Streptavidin (0.5 mg ml^−1^) (Sigma), dissolved in 3% trehalose. For the control line goat monoclonal anti-rabbit IgG was used (1 mg ml^−1^ in 3% trehalose). As a control gold conjugate, 1 mg of polyclonal rabbit IgG per 100 ml gold particles was prepared as described before. The LFA strips (width 4 mm) were assembled and put into plastic cassettes.

### Comparison between *Tco*PYK ELISA, *Tco*PYK LFA and microscopy as survey tools on *T*. *congolense* infected mice

C57BL/6 mice (n = 9) were inoculated intraperitoneally with 5000 *T*. *congolense* (Tc13) parasites. The mice were bled at different times post-infection (day 3, 7, 11, 15, 19, 23, 27, 31 and 35). During each time point 100 *μ*l of whole blood was collected from the tail of each individual using heparin-coated capillary tubes. Two *μ*l of blood was used to follow-up mice parasitaemia by diluting the sample 100-fold (during high parasitaemia periods) or 50-fold (low parasitaemia periods) in RPMI medium and counting the parasites under the light microscope. The rest of the blood was centrifuged at 1500 g for 10 minutes. Plasma was collected and tested in the ELISA and the LFA. For the ELISA, 30 *μ*l of plasma was diluted in 60 *μ*l of PBS-T, while for the LFA 20 *μ*l plasma was diluted in 40 *μ*l PBS-T containing 1% methylcellulose (Sigma) per sample and applied onto the sample pad. After maximum 20 minutes, the result could be read from the test line. ELISA, LFA and microscopy results were compared measuring the inter-rater reliability between tests through the Cohen’s kappa coefficient^81^.

### *Tco*PYK sandwich ELISA and LFA during *T*. *congolense* treatment follow-up in mice

The PYK antigaenemia during the course of a *T*. *congolense* infection in mice before and after diminazene aceturate treatment (Berenil by CEVA) was assessed using the Nb44-Nb44/Nb42 sandwich ELISA and the *Tco*PYK LFA. C57BL/6 mice were divided in three groups of six individuals each. In each group, five mice were inoculated intraperitoneally with 5000 *T*. *congolense* (Tc13) parasites. The remaining mouse in each group was used as a naive negative control. All individuals from group 1 and 2 were treated with Berenil (20 g per kg animal) administered intraperitoneally at day 6 (group 1) and day 14 (group 2) post-infection, while group 3, remained untreated during the course of infection. Mice were bled at different times points before and after infection (day −1, 4, 7, 10, 13, 16, 19 and 22). The bleeding, ELISA, microscopy and LFA analysis are performed as described before.

### *Tco*PYK LFA to diagnose cattle experimentally infected with *T*. *congolense*

Frozen bovine plasma samples (−80 °C) were kindly provided by Clinvet (Bloemfontein, South-Africa). The original blood samples were collected at different time points during an experimental trypanosome infection whereby 22 Holstein cows were injected with *T*. *congolense* Savannah-type KONT 2/133 (isolated in 2004 from naturally infected cattle in Cameroon, stabilate from Belgian Tropical Institute of Medicine) and 22 Holstein cows were injected with *T*. *vivax* STIB 719 (isolated as Y486 strain in 1976 from naturally infected cattle in Nigeria, stabilate from Swiss Tropical and Public Health Institute). Pre-infected samples served as the naive control samples. Of each sample, 20 *μ*l plasma was diluted in 40 *μ*l PBS-T containing 1% methylcellulose (Sigma) and applied onto the sample pad of the LFA. After maximum 20 minutes, the result could be read visually from the test line.

## Electronic supplementary material

Supplementary Information
