# Supplementary material for: Development of a Nanobody-based lateral flow assay to detect active Trypanosoma congolense infections
Source: Sci Rep. 2018 Jun 13;8:9019. doi: 10.1038/s41598-018-26732-7 (PMC5998082; doi:10.1038/s41598-018-26732-7)
Supplement: Supplementary file 1 — Supplementary Information [file 41598_2018_26732_MOESM1_ESM.pdf]

Supplementary Information for

Development of a Nanobody-based lateral flow assay to detect active *Trypanosoma congolense* infections

Joar Pinto[1,2,+], Julie Goossens[1,2,+], Jianzu Ding[3], Zeng Li[1,2], Shaohong Lu[3], Didier Vertommen[4], Peter Naniima[&], Rui Chen[3], Serge Muyldermans[1], Yann G.-J. Sterckx[1,2,‡], Stefan Magez[1,2,5,‡]

[1]Research Unit for Cellular and Molecular Immunology (CMIM), VUB, Pleinlaan 2, B-1050 Brussels, Belgium

[2]Structural Biology Research Center (SBRC), VIB, Pleinlaan 2, B-1050 Brussels, Belgium

[3]Institute of Parasitic Diseases, Zhejiang Academy of Medical Sciences, Hangzhou 310013, Zhejiang, China

[4]Department of Metabolism and Hormones, de Duve Institute, Université Catholique de Louvain, Avenue Hippocrate 75, B-1200 Brussels, Belgium

[5]Ghent University Global Campus, Songdomunhwa-Ro 119, Yeonsu-Gu, 406-840 Incheon, South Korea

[*]Corresponding author: stefan.magez@vub.be

[+]these authors contributed equally to this work and should be considered joint first authors

[‡]these authors contributed equally to this work and should be considered joint senior authors

[&]Current address: Institute of Virology, Structural Virology Group, Medizinische Hochschule Hannover, Carl-Neuberg-Strasse 1, 30625 Hannover, Germany

This document includes:

Supplementary Methods

5 Supplementary Figures and their respective legends

Supplementary Methods

Nb library construction and phage display onto *T. congolense* secretome

A male alpaca was injected once a week for a period of 6 weeks with 100 μg of *T. congolense* IL3000/IL1180 secretomes mixed in equal ratio in GERBU adjuvant (GERBU Biotechnik GmbH, Germany). 50 ml of blood was collected four days after the last immunisation. Lymphocytes from peripheral blood were isolated using lymphoprep (Stemcell technologies) from which the messenger RNA (mRNA) was isolated for cDNA synthesis. The coding sequences of the variable region from the single-domain antibodies were amplified in a two-step nested PCR using specific primers as previously described[1]. Amplicons were cloned into the phage display vector pHEN4[2] and transformed into *E. coli* TG1 electrocompetent cells (Lucigen. USA). Phage particles expressing the immune VHH repertoire were obtained after transfection of the library with 1x10^{12} M13K07 helper phages particles. 1x10^{11} phages expressing the VHH repertoire were used to pan against *T. congolense* secretome (10 μg per well of a 96-well NUNC plate, Thermo scientific). Elution of phages particles was carried out with 100 mM triethylamine (pH $\sim$10.0) followed by neutralisation with 100 μl 1 M Tris (pH 8.2). Eluted phages were amplified by infecting fresh *E. coli* TG1 cells in the exponential phase. Three rounds of panning were performed subsequently on distinct samples: *T. congolense* IL3000 secretome (round 1), *T. congolense* IL1180 secretome (round 2), and *T. congolense* Tc13 soluble proteome (round 3). Screening and selection of colonies carrying Nb fragments targeting the parasite's secretome was assessed through an ELISA on the periplasmic extract (PE-ELISA), which was performed as described[1]. The experimental details of the PE-ELISA are given in the next section. Colonies that scored positive in the PE ELISA were subjected to plasmid extraction. Thereafter, the gene sequences of those binders were analysed in order to identify unique Nb sequences using Clone Manager Main Workbench 6.9 software (QIAGEN bioinformatics).

Nb cloning, production, and purification

In brief, the Nb-encoding genes were amplified by PCR. Generated fragments were double digested using *Pst*I/*Eco*91I restriction enzymes followed by ligation into the following expression vectors using T4 DNA ligase (Roche): pHEN6c, pMECS and pBAD[3,4]. The pHEN6c vector equips the Nbs with a C-terminal hexahistidine (His) tag, while the pMECS vector allows production of the Nbs in fusion with a C-terminal HA-tag followed by a His-tag. The pBAD vector equips the Nbs with a C-terminal biotin acceptor domain (so-called AVI-tag[5]). All Nb plasmids were transformed into *E. coli* DH5α WK6 cells. In case of the pBAD constructs, *E. coli* WK6 was co-transformed with a plasmid containing the *E. coli* biotin-ligase BirA to allow *in vivo* biotinylation onto the AVI-tag. Protein production and purification of the His-tagged and biotinylated Nbs was performed as described[1,6], respectively.

Inserting a $GGGGS_4$ linker in between two Nb44 gene copies generated the bivalent Nb44-Nb44 construct, which was produced and purified as described[4].

Production and purification of *Tco*PYK, *Tbr*PYK and *Lme*PYK

After transformation, single colonies were pre-cultured overnight at 37°C in 10 ml LB media supplemented with 100 μg ml^{-1} ampicillin. One ml of each pre-culture was used to inoculate a flask containing 330 ml of 2xTY media supplemented with 2% glucose, 2 mM $MgCl_2$ and 100 μg ml^{-1} ampicillin. Cells were grown at 37°C with aeration (220 rpm) until they reached exponential phase ($O.D._{600nm}$ $\sim$0.8). Protein production was induced by addition of 1 mM isopropyl β-D-1-thiogalactopyranoside (IPTG). After induction, the cultures were incubated overnight at 20°C with aeration (160 rpm). Cells were harvested by centrifugation (8 min, 11325 g, 4°C). Bacterial pellets were resuspended in 17 ml lysis buffer (50 mM Tris-HCl, 500mM NaCl, 30 μg ml^{-1} AEBSF, 1 μg ml^{-1} leupeptin, 0.1 mM EDTA) and aliquoted in volumes of $\sim$30 ml. The aliquots were flash-frozen using liquid nitrogen and stored at -20°C. Prior to purification, aliquots were thawed on ice. Cells were lysed using a sonicator (Ultrasonic disintegrator MSE Soniprep 150; 5 sonication cycles of 1 min at 15 microns amplitude with a 2 min pause between each cycle) and the cell lysate was centrifuged (30 min, 25400 g, 4°C). The supernatant was collected and filtered (0.45 μm). Protein purification was performed on an AKTA Prime Platform (GE Healthcare) using IMAC and SEC. A 5 ml HisTrap HP nickel-sepharose column (GE Healthcare) was equilibrated with buffer A (50 mM Tris-HCl, 500 mM NaCl, pH 8.0) for at least five column volumes. The sample was loaded on the column using the same buffer at a flow rate of 1 ml min^{-1}. After loading, the column was further washed with 5 column volumes of the same buffer. The target protein (*Tco*PYK, *Tbr*PYK or *Lme*PYK) was then eluted by a linear gradient of buffer B (50 mM Tris-HCl, 500 mM NaCl, 1 M imidazole, pH 8.0) over 20 column volumes. The fractions containing the target protein were pooled and concentrated to a final volume of 2 ml for the subsequent SEC step on a Superdex 200 16/60 column (GE Healthcare), which was pre-equilibrated with at least one column volume of buffer C (20 mM Tris-HCl, 150 mM NaCl, pH 7.2). The sample was eluted at a flow rate of 1 ml min^{-1}. Fractions containing the target protein were pooled and stored at 4°C. Each of the purification steps was monitored by SDS-PAGE and Western blot under reducing conditions.

ELISA

PE ELISA. From the three consecutive rounds of panning, 95 colonies were randomly selected and grown in 1 ml cultures expressing the individual Nb clones. 200 μl of periplasmic extract (PE) from each colony was collected, from which 100 μl was used for the PE ELISA. In this experimental set-up, *T. congolense* IL3000 secretome was employed as capturing material (10 μg per well) and the PEs were the samples to be tested for secretome recognition. Mouse monoclonal anti-hemaglutinin-biotin antibody (anti-HA-B, Sigma) was employed as a primary detection reagent (diluted to a concentration of 0.5 μg ml^{-1} in 3% blocking buffer).

Sandwich ELISA pairing assay. Five Nb clones were retrieved from the PE-screening and were tested for their potential to form a sandwich ELISA pair using the *T. congolense* secretome as crude antigen. In this experimental set-up, the capturing and detecting Nbs were His-tagged and HA-His-tagged, respectively The capturing Nbs (Nb20H, Nb26H, Nb42H, Nb44H, Nb45H) were coated at a concentration of 2 μg ml^{-1}. *T. congolense* IL3000 secretome diluted in PBS (final concentration of 10 μg ml^{-1}) was added to the wells at a volume of 100 μl per well. The detection Nbs (Nb20HA, Nb26HA, Nb42HA, Nb44HA, Nb45HA) were added at a concentration of 1 μg ml^{-1}.

Specificity of the Nb44/Nb42 sandwich ELISA on trypanosome-infected mice sera. The specificity of the Nb44/Nb42 sandwich ELISA was assessed using serum samples from mice infected with different *T. congolense* strains and other closely related trypanosomatids. The infected mice were bled on 7 days post-infection. The serum aliquots were pooled and diluted two times in PBS before being evaluated with the above-mentioned ELISA set-up using Nb44H as capturing material (coated at 5μg ml^{-1} per well) and Nb42HA as primary detection reagent (applied at a concentration of 1μg ml^{-1} per well).

Nb44/Nb42 sandwich ELISA on recombinant PYKs. The specificity of Nb44/Nb42 sandwich ELISA for *T. congolense* was examined using recombinant PYKs. The capturing Nb, Nb44H, was coated at 5 μg ml^{-1} per well. The *Tco*PYK, *Tbr*PYK and *Lme*PYK samples were added to each well (diluted to a concentration of 1 μg ml^{-1} in PBS). Nb42HA was employed as a primary detection reagent (applied at a concentration of 5 μg ml^{-1} per well).

Overcoming the plasma matrix effect on the Nb44/Nb42 sandwich ELISA. Mouse plasma was spiked with recombinant *Tco*PYK (3 μg ml^{-1}) and either used as such or diluted 3-fold in PBS-based buffers containing different amount of salt (100 mM and 300 mM NaCl) or non-ionic detergents (0.5% and 1.0% Tween20). The capturing Nb, Nb44H, was coated at 5 μg ml^{-1} per well. Nb42HA was employed as a primary detection reagent (applied at a concentration of 1 μg ml^{-1} per well).

Nb44/Nb42 ELISA checkerboard titration. The amount of capturing (Nb44H) and detecting (Nb42HA) Nbs required to obtain the highest signal intensity in the sandwich ELISA was determined by checkerboard titration. Briefly, a two-fold serial dilution of Nb44H (initial concentration 5 μg ml^{-1}) was prepared in PBS. Each Nb44H dilution was coated in triplicate. Recombinant *Tco*PYK was added to each well (diluted to a concentration of 1 μg ml^{-1} in PBS). Nb42HA was titrated in the same way as Nb44H (initial concentration of 0.25 μg ml^{-1}).

Increasing the LoD of the *Tco*PYK sandwich ELISA. The initial *Tco*PYK sandwich ELISA set-up was modified in order to improve the sensitivity of the assay. First the bivalent capturing Nb44-Nb44 and the biotinylated detection Nb42 (Nb42B) were titrated in PBS in a checkerboard fashion in order to determine the optimal concentration of each Nb to obtain the highest signal intensity as performed previously. Starting concentrations for Nb44-Nb44 and biotinylated Nb42 were 6 μg ml^{-1} and 0.25 μg ml^{-1}, respectively. Mouse plasma was spiked with recombinant *Tco*PYK (1 μg ml^{-1}) and used to generate a 2-fold dilution series. Each sample in this series was again diluted 3-fold in PBS-T to reduce matrix effects. Two ELISA plates were tested in parallel. The first plate contained Nb44H as capturing agent (5 μg ml^{-1} coated per well) and streptavidin-HRP as secondary antibody (0.5 μg ml^{-1} in protein free blocking buffer). The second plate contained Nb44-Nb44 as capturing agent (5μg ml^{-1} coated per well) and streptavidin poly-HRP as secondary antibody (1 μg ml^{-1} in protein free blocking buffer). In both plates, Nb42B was employed as a primary detection reagent (concentration 1 μg ml^{-1}).

Surface plasmon resonance

Surface plasmon resonance (SPR) experiments were performed on a BIAcore T200 system (GE Healthcare). The interactions between the two Nbs (Nb42 and Nb44) and *Tco*PYK were analysed on a CM5 chip. The Nbs were immobilised in flow cell 2 using the following procedure. Using a flow rate of 5 μl min^{-1} the carboxylated dextran matrix was activated by a 7-min injection of a solution containing 0.2 M N-ethyl-N'-(3-diethylamino)propyl carbodiimide (EDC) and 0.05 M N-hydroxysuccinimide (NHS). A Nb solution of 1 μg ml^{-1} (50 mM sodium acetate pH 5.0) was subsequently injected until the desired amount of protein was immobilised (approx. 100 R.U.). The surface immobilisation was then blocked by a 7-min injection of 1 M ethanolamine hydrochloride. The surface in flow cell 1 was used as a reference and treated only with EDC, NHS and ethanolamine. *Tco*PYK was dialysed into the running buffer (20 mM HEPES, 150 mM NaCl, 0.005% Tween, 3.4 mM EDTA, pH 7.4) prior to data collection.

For Nb42, sensorgrams for different concentrations of *Tco*PYK expressed as monomer concentrations (0.05 nM, 0.10 nM, 0.25 nM, 0.50 nM, 1.00 nM, 2.50 nM, 5.00 nM, 7.50 nM, 10.00 nM, 12.50 nM, 15.00 nM, 20.00 nM, 25.00 nM, 50.00 nM) plus a 0 concentration (injection of running buffer) were collected at a flow rate of 30 μl min^{-1} and a data collection rate of 1

Hz. Analyte injections were performed with association and dissociation phases of 360 s and 600 s, respectively. This was followed by a 15 μl pulse injection of regeneration buffer (10 mM glycine pH 2.0). Prior to data analysis, reference and zero concentration data were subtracted from the sensorgrams. The data collected for the Nb42-*Tco*PYK interaction were analysed with a 1:1 Langmuir binding model.

For Nb44, data for the interaction with *Tco*PYK was collected in the format of a kinetic titration[7] due to the absence of a suitable regeneration condition. Sensorgrams were collected at four different concentrations (1/3 serial dilution starting from 333.33 nM) plus a 0 concentration (injection of running buffer) at a flow rate of 30 μl min^{-1} and a data collection rate of 10 Hz. Analyte injections were performed with association phases of 120 s and a dissociation phase of 240 s. Prior to data analysis, reference and zero concentration data were subtracted from the sensorgrams. The data were analysed with a 1:1 Langmuir binding model[7].

Supplementary Figures

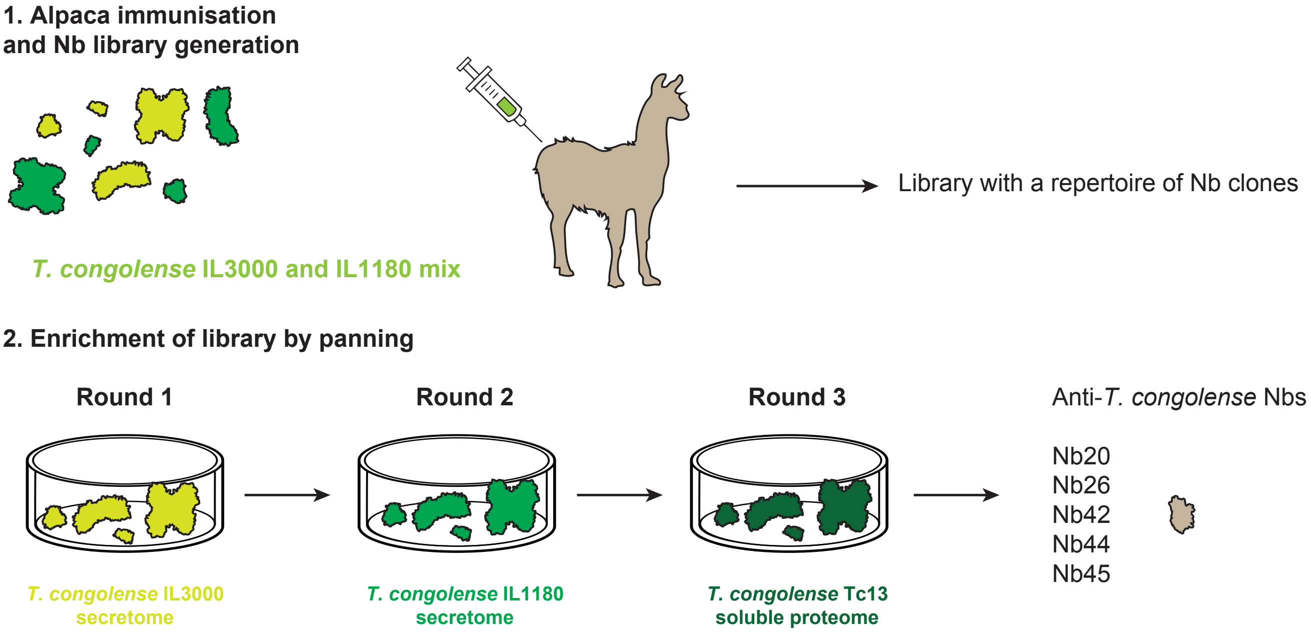

Supplementary Figure S1. Schematic illustration of the Nb library generation, panning and screening strategy. The Nb library was generated from an alpaca that was immunized with a mixture of the secretomes of *T. congolense* strains IL1180 and IL3000. Three rounds of panning were performed subsequently on distinct samples: *T. congolense* IL3000 secretome (round 1), *T. congolense* IL1180 secretome (round 2), and *T. congolense* Tc13 soluble proteome (round 3).

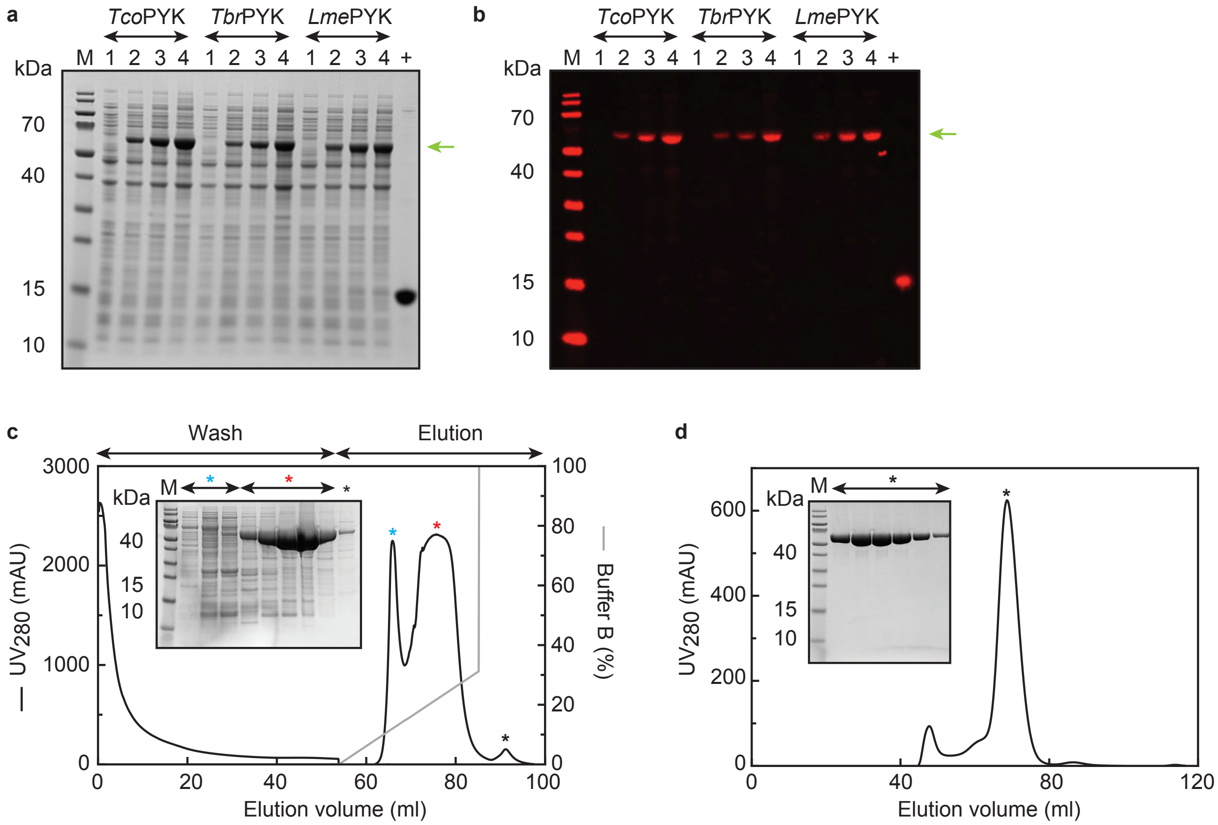

Supplementary Figure S2. Overview of the production and purification of *Tco*PYK, *Tbr*PYK, and *Lme*PYK. (**a-b**) Recombinant production of *Tco*PYK, *Tbr*PYK, and *Lme*PYK in *E. coli* BL21(DE3). Samples of the bacterial culture were taken before induction (Lane '1'), 3 hours after induction (Lane '2'), 6 hours after induction (Lane '3') and after overnight incubation after induction of gene expression (Lane '4') and analysed by SDS-PAGE (**a**) and Western blot (**b**). The bands corresponding to His-tagged *Tco*PYK, *Tbr*PYK, and *Lme*PYK are indicated by the black arrow (MM = 56 kDa). Lane '+', His-tagged Nb as positive control. Lane 'M', Prestained Protein Molecular Weight Marker (ThermoFischer Scientific). (**c-d**) Purification of *Tco*PYK by IMAC (**c**) followed by gel filtration (**d**). The insets in both panels show an SDS-PAGE analysis of the collected fractions (indicated by the asterisk '*'). The purifications of *Tbr*PYK and *Lme*PYK are performed according to the same protocol and yield similar results.

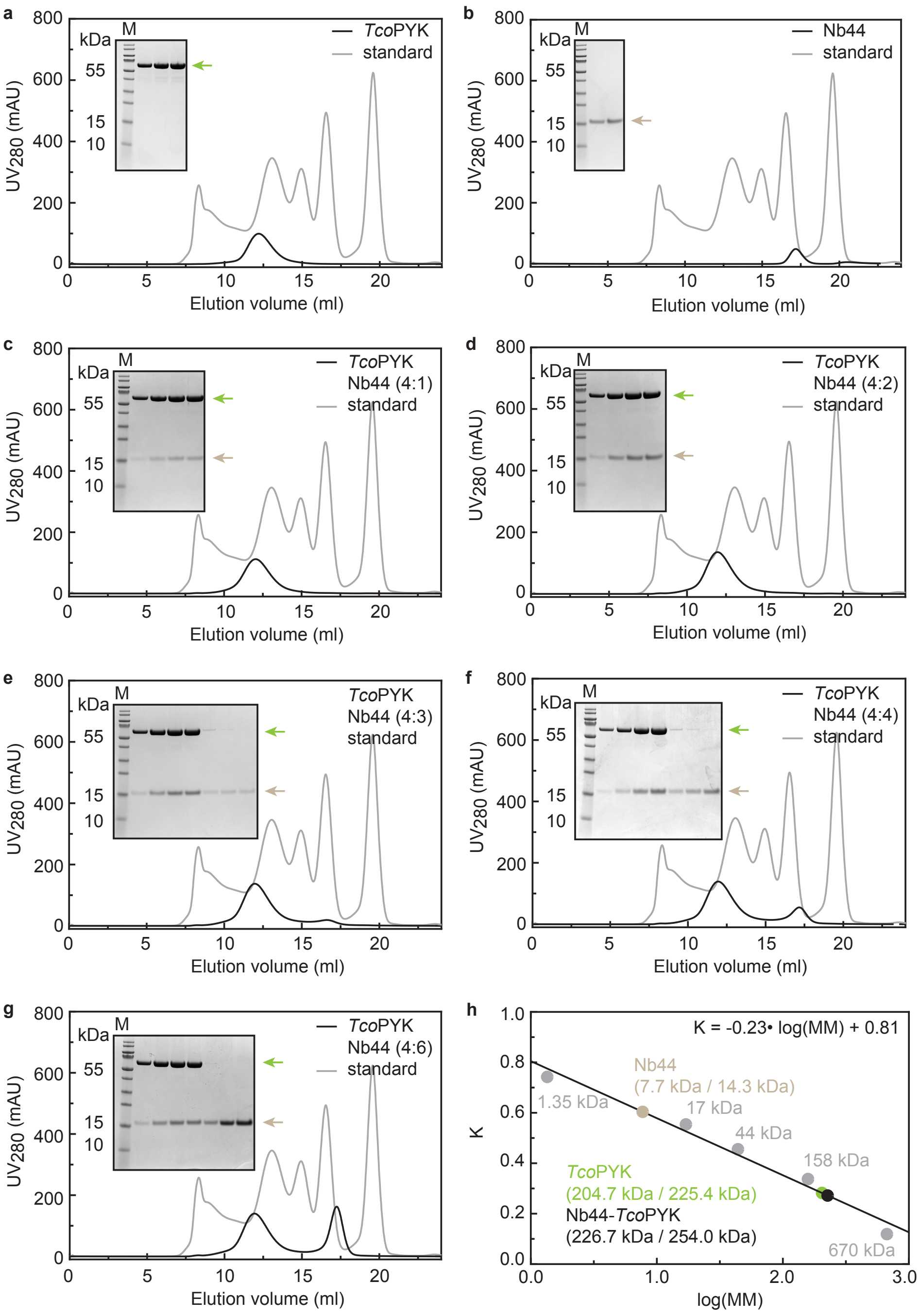

Supplementary Figure S3. Investigation of the stoichiometry of the Nb44-*Tco*PYK complex by analytical SEC. (a-g) Analytical SEC on the purified *Tco*PYK (**a**), Nb44 (**b**) and samples containing *Tco*PYK and Nb44 mixed at different molar ratios: 4:1 (**c**), 4:2 (**d**), 4:3 (**e**), 4:4 (**f**), and 4:6 (**g**). All experiments were performed on a Superdex 200 HR 10/30 column. The black and grey traces represent the chromatograms of the different protein samples and the BioRAD gel filtration standard, respectively. In all figures, the inset shows an SDS-PAGE analysis of the elution peaks. *Tco*PYK (MM = 56.2 kDa) and Nb44 (MM = 14.3 kDa) are indicated by the green and brown arrows, respectively. Lane M, Prestained Protein Molecular Weight Marker (ThermoFischer Scientific). (**h**) The calibration of the Superdex 200 HR 10/30 column that allows estimation of molecular mass based on the sample's elution volume. The values between brackets indicate the estimated versus the theoretical molecular mass of the sample under investigation.

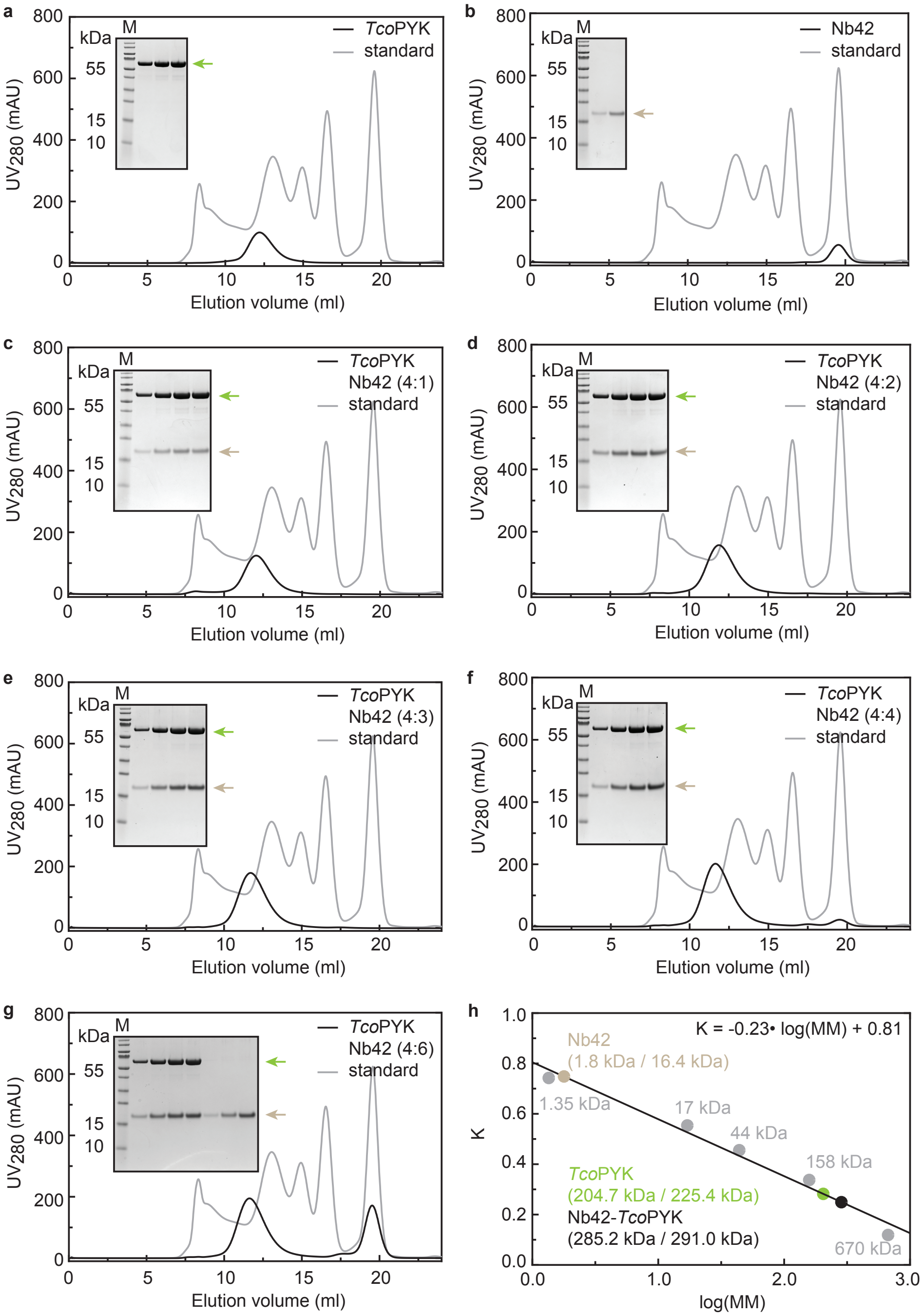

Supplementary Figure S4. Investigation of the stoichiometry of the Nb42-*Tco*PYK complex by analytical SEC. (a-g) Analytical SEC on the purified *Tco*PYK (**a**), Nb42 (**b**) and samples containing *Tco*PYK and Nb42 mixed at different molar ratios: 4:1 (**c**), 4:2 (**d**), 4:3 (**e**), 4:4 (**f**), and 4:6 (**g**). All experiments were performed on a Superdex 200 HR 10/30 column. The black and grey traces represent the chromatograms of the different protein samples and the BioRAD gel filtration standard, respectively. In all figures, the inset shows an SDS-PAGE analysis of the elution peaks. *Tco*PYK (MM = 56.2 kDa) and Nb42 (MM = 16.4 kDa) are indicated by the green and brown arrows, respectively. Lane M, Prestained Protein Molecular Weight Marker (ThermoFischer Scientific). (**h**) The calibration of the Superdex 200 HR 10/30 column that allows estimation of molecular mass based on the sample's elution volume. The values between brackets indicate the estimated versus the theoretical molecular mass of the sample under investigation.

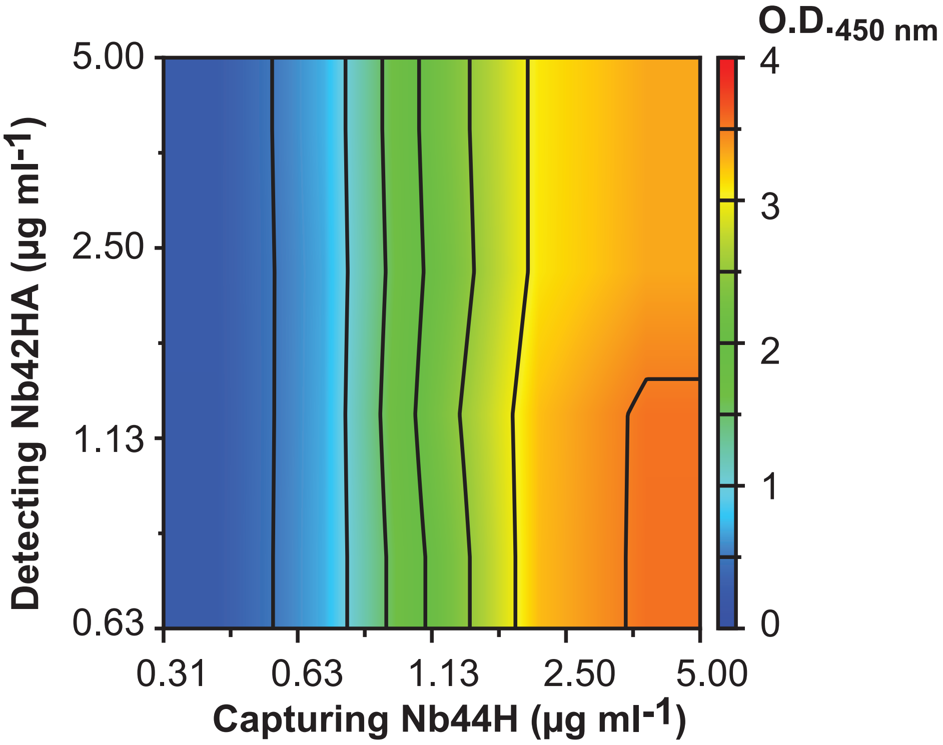

Supplementary Figure S5. A heat map readily identifies the optimal practical set-up to conduct the Nb44/Nb42 ELISA. Using a checkerboard system, varying amounts of capturing and detecting Nbs were employed to identify those conditions yielding the highest ELISA signal. The details of this approach are described in the Methods section of this Supplementary Information file.

References

1. Conrath, K. E. *et al.* Beta-lactamase inhibitors derived from single-domain antibody fragments elicited in the *Camelidae*. *Antimicrob Agents Chemother* **45**, 2807–12 (2001).
2. Arbabi Ghahroudi, M., Desmyter, A., Wyns, L., Hamers, R. & Muyldermans, S. Selection and identification of single domain antibody fragments from camel heavy-chain antibodies. *FEBS Lett* **414**, 521–6 (1997).
3. Vincke, C. *et al.* Generation of single domain antibody fragments derived from camelids and generation of manifold constructs. *Methods Mol Biol* **907**, 145–76 (2012).
4. Conrath, K., Lauwereys, M., Wyns, L. & Muyldermans, S. Camel single-domain antibodies as modular building units in bispecific and bivalent antibody constructs. *J Biol Chem* **276**, 7346–50 (2001).
5. Schatz, P. J. Use of peptide libraries to map the substrate specificity of a peptide-modifying enzyme: a 13 residue consensus peptide specifies biotinylation in *Escherichia coli*. *Biotechnol. (N Y)* **11**, 1138–43 (1993).
6. Zhu, M., Gong, X., Hu, Y., Ou, W. & Wan, Y. Streptavidin-biotin-based directional double Nanobody sandwich ELISA for clinical rapid and sensitive detection of influenza H5N1. *J Transl Med* **12**, 352 (2014).
7. Karlsson, R., Katsamba, P. S., Nordin, H., Pol, E. & Myszka, D. G. Analyzing a kinetic titration series using affinity biosensors. *Anal Biochem.* **349**, 136–47 (2006).
